# Glu-Urea-Lys Scaffold Functionalized Superparamagnetic Iron Oxide Nanoparticles Targeting PSMA for In Vivo Molecular MRI of Prostate Cancer

**DOI:** 10.3390/pharmaceutics14102051

**Published:** 2022-09-26

**Authors:** Wei Zhou, Jiandong Huang, Qingwei Xiao, Shunmin Hu, Shijia Li, Jie Zheng, Zhiyun Du, Jiangling Peng, Huixiong Chen

**Affiliations:** 1 School of Biomedical and Pharmaceutical Sciences, Guangdong University of Technology, 100 Waihuanxi Road, Guangzhou 510006, China; 2 Laboratory of Pharmacologic and Toxicologic Chemistry and Biochemistry, UMR 8601 CNRS, Paris Descartes University, 75006 Paris, France

**Keywords:** prostate cancer, PSMA, molecular imaging, MRI contrast agent, SPIONs

## Abstract

The prostate specific membrane antigen (PSMA), extensively overexpressed on prostate cancer (PCa) cell surface, has been validated as a diagnostic biomarker for PCa. However, insufficient attention has been paid to the development of PSMA-specific probes loaded with small chemical molecules for the in vivo molecular imaging of PCa. In this study, we innovatively labelled superparamagnetic iron oxide nanoparticles with a PSMA-targeting Glu-Urea-Lys scaffold. An optimized synthetic route was developed to offer a physiochemically stable probe. The probe demonstrated high binding affinity (0.38 ± 0.08 μg(Fe)/mL) and binding specificity to PSMA expressed on prostate cancer cell surface in vitro. In a xenograft PCa mouse model, significant negative contrast of the implanted prostate cancer xenograft could be specifically observed by MRI 6 h after tail vein injection of the tracer (Fe, 20 mg/kg), exhibiting its potential to exclusively enhance magnetic resonance detection of PCa.

## 1. Introduction

Prostate cancer (PCa) is one of the most commonly diagnosed malignancies, affecting approximately 1.3 million people worldwide in 2018 [1]. It has already become the most frequent cancer among men and the second leading course of cancer-related deaths in the United States [2]. Early diagnostic strategies (including prostate-specific antigen screening tests) and the treatment of PCa markedly reduced the incidence of late-stage metastasis and mortality [3,4]. Therefore, there is a pressing demand of imaging modalities for PCa [5]. Molecular imaging techniques have been integrated into the management of patients with PCa for decades [6]. Molecular targeted diagnostic agents capable of preferential delivery to the intended site have dramatically improved PCa diagnosis [7]. Molecular imaging probes that bind with PCa specific biomarkers could significantly improve sensitivity and specificity for PCa diagnosis.

The integral membrane protein prostate-specific membrane antigen (PSMA) is excessively expressed in primary and metastatic PCa. Notably, the expression level of PSMA is positively correlated with higher PCa grade [8], and could serve as an important indicator for PCa staging and treatment management [9]. This characteristic endows PSMA with particular value since it has the potential to serve as an early indicator of tumor progression [10]. The accuracy of imaging agents based on PSMA-ligand binding is superior to that of traditional imaging tracers such as choline [11]. It has become increasingly recognized as a validated target for developing diagnostic or therapeutic agents of PCa. Over the past two decades, many high-affinity antibodies such J415, J533 and J591 [12], and small molecule tracers such as ^99m^Tc-MIP-1404/-1405 [13], [^18^F]-DCFBC [14] have been extensively and successfully adopted in developing PCa molecular imaging tracers [10]. PSMA-targeting small molecular tracers processing Glu-Urea-Lys cores, many of which have been widely explored and exploited in PSMA specific tracers for PCa radiodiagnosis [15,16], exhibited several favorable characteristics, such as high binding affinity, promising target specificity, rapid distribution, and faster blood clearance [17].

PSMA specific molecular imaging modalities for prostate cancer mainly include magnetic resonance imaging (MRI) and positron emission tomography (PET). Though PSMA PET tracers for PCa PET imaging, including ^18^F-DCFPyL and ^68^Ga-PSMA-11, have already been approved by FDA [18,19], accessibility of PET diagnosis to general patients remains challenging. Thus, the low threshold of preparation and utilization of imaging agents offers MRI with additional advantages. Besides, owing to modern signal amplification strategies, MR may offer increased sensitivity for molecular imaging.

Recently, much effort has been focused on the development of novel molecular contrast agents for MRI of PCa [20,21,22,23,24]. Superparamagnetic iron oxide nanoparticles (SPIONs) have recently received substantial attention due to their favorable negative contrasting effect of PCa lesions with contiguous healthy tissues [25]. Moreover, SPIONs could readily serve as ideal carriers for various targeting motifs, including small ligands, monoclonal antibodies, polypeptides, and nucleic acid aptamers. In earlier studies, antibodies were commonly adopted for the construction of PSMA-targeting SPIONs [26,27].

However, as far as we know, less attention has been paid to PSMA-specific SPIONs labeled with small chemical molecules. In this study, we attempted to identify target-specific, bio-compatible tracer for molecular MRI of PCa. To this end, we innovatively designed superparamagnetic iron oxide nanoparticles mounted with a PSMA-targeting Glu-Urea-Lys scaffold, a small chemical molecule presumably with better tissue penetration. The in vitro binding affinity and selectivity were evaluated, and in vivo MR imaging was conducted in a mouse model of PCa xenograft tumor.

## 2. Materials and Methods

### 2.1. General Materials

Solvents and chemicals of analytical grade or above were obtained from commercial sources without further purification. All in vitro experiments were repeated in triplicate or more, for statistical analysis. The reactions were monitored by thin layer chromatography (TLC) using Qing Dao Hai Yang GF254 silica gel plates visualized with ultraviolet (UV) light (254 nm), iodine steam or phosphomolybdic acid (PMA), and column chromatography was performed using silica gel (200–300 mesh).

### 2.2. Chemistry

#### 2.2.1. Di-*Tert*-Butyl L-Glutamate (**1**)

A solution of L-glutamate (5 g, 1.0 eqv) in *tert*-butyl acetate (100 mL) was stirred in an ice bath for 5 min, then 70% perchloric acid solution (2.0 eqv) was slowly added at room temperature. Stirred overnight, the reaction was cooled to 0 °C, and extracted with 0.5 M cold hydrochloric acid (2 × 30 mL), and the aqueous phase was collected. The sodium carbonate was slowly added to the aqueous phase and stirred (pH = 8~10), extracted (CH_2_Cl_2_) and dried (Na_2_SO_4_). The filtered liquid was concentrated in vacuo, compound **1** was obtained as a colorless or pale yellow transparent viscous liquid, yield: 47.6%. ^1^H NMR (400 MHz, CDCl_3_) *δ*:3.50–3.40 (m, 1H), 3.03 (s, 3H), 2.37 (t, *J* = 7.5 Hz, 2H), 2.02 (d, *J* = 10.3 Hz, 1H), 1.90–1.76 (m, 1H), 1.47 (t, *J* = 8.9 Hz, 18H). ^13^C NMR (101 MHz, CDCl_3_) *δ*:173.95, 172.61, 81.73, 80.64, 54.11, 31.82, 29.44, 28.09, 28.03. HR-MS(ESI) *m*/*z*: Calcd for C_13_H_25_NO_4_{[M + H]^+^} 260.18524, found 260.18563, ${\left[\alpha \right]}_{D}^{20}$ = 22.86 (C = 0.687, CH_3_OH).

#### 2.2.2. N^6^-Fmoc-N^2^-Boc-L-Lysine (**2**)

The synthesis of compound **2** was carried out in two steps. Step 1: a solution of sodium hydrogencarbonate (17 g) in water (50 mL) was stirred and then *L*-lysine hydrochloride (10 g) was added. After 15 min, anhydrous copper (II) sulfate (7 g) was added to the reactor for 1 h, and then 9-fluorenylmethyl-N-succinimide carbonate (Fmoc-OSU, 22.1 g) and THF (100 mL) were added. After stirring for 14 h, sodium hydrogencarbonate (100 mL) was added and stirred for 3 h. Next, water (50 mL) and methanol (20 mL) were added and stirred for 5 h, then ethyl acetate (50 mL) was added and stirred for 30 min. Finally, the mixture was filtered, the residue was washed with water to be colorless. The copper complex intermediate of *N*^6^-Fmoc-*L*-lysine was obtained after drying in a vacuum drying oven. Step 2: the dried intermediate (10 g) was dissolved in THF/water (1:1). Anhydrous sodium carbonate (10 g) and 8-hydroxyquinoline (5 g) were added in batches. After stirring at room temperature for 2 h, Boc anhydride (6 g) was added. After 5 h, the filtrate from the mixture was cooled to below 5 °C. The pH was adjusted to 2 to 3 with hydrochloric acid, and the crude product was obtained by extraction, and purification by column chromatography on silica gel. Compound **2** was a yellow viscous liquid with combined total yield: 65%. ^1^H NMR (400 MHz, CDCl_3_) *δ*: 7.67 (t, *J* = 6.9 Hz, 2H), 7.51 (d, *J* = 7.2 Hz, 2H), 7.32 (t, *J* = 7.4 Hz, 2H), 7.23 (t, *J* = 7.3 Hz, 2H), 6.08 (s, 0.4H), 5.58 (s, 0.2H), 5.13 (s, 0.6H), 4.85 (s, 0.8H), 4.27 (dd, *J* = 62.5, 28.3 Hz, 6H), 3.11 (d, *J* = 5.9 Hz, 2H), 1.89–1.22 (m, 13H). ^13^C NMR (101 MHz, CDCl_3_) *δ*: 176.46, 156.74, 155.88, 143.97, 127.70, 127.08, 125.09, 119.98, 80.17, 66.70, 53.27, 47.27, 40.63, 32.05, 29.35, 28.36, 22.44. HR-MS(ESI) *m*/*z*: Calcd for C_26_H_32_N_2_O_6_{[M-H]^−^} 467.21884, found 467.21876.

#### 2.2.3. *Tert*-Butyl N^6^-Fmoc-L-Lysinate (**3**)

Compound **2** (1.0 eqv, 2 g) was dissolved in *tert*-butyl acetate (50 mL) and stirred at low temperature for 5 min. Next, 70% perchloric acid (2.0 eqv) was added dropwise. After stirring at room temperature overnight, the reaction was cooled to 0 °C, and NaOH aqueous solution (2 M) was carefully added (PH = 10). Immediately extracted with ethyl acetate, dried by anhydrous sodium sulfate, filtered the crude product was then purified by column chromatography on silica gel to afford compound **3** as a colorless transparent viscous liquid with yield 70%. ^1^H NMR (400 MHz, CDCl_3_) *δ*: 7.70 (d, *J* = 7.5 Hz, 2H), 7.57 (d, *J* = 7.3 Hz, 2H), 7.34 (t, *J* = 7.4 Hz, 2H), 7.26 (dd, *J* = 9.9, 4.7 Hz, 2H), 4.39–4.24 (m, 1H), 4.15 (d, *J* = 8.3 Hz, 0H), 4.00 (t, *J* = 5.9 Hz, 1H), 3.12 (s, 1H), 1.94 (d, *J* = 5.2 Hz, 1H), 1.46 (d, *J* = 30.9 Hz, 14H). ^13^C NMR (101 MHz, CDCl_3_) *δ*: 171.28, 168.49, 156.96, 143.99, 141.24, 127.66, 127.11, 125.26, 125.08, 119.89, 84.69, 66.81, 60.45, 54.16, 47.12, 40.34, 29.97, 29.13, 27.82, 21.72, 21.07, 14.21. HR-MS(ESI) *m*/*z*: Calcd for C_25_H_32_N_2_O_4_{[M + H]^+^} 425.24310, found 425.24348, ${\left[\alpha \right]}_{D}^{20}$ = 7.9 (C = 1, CH_3_OH).

#### 2.2.4. (*Tert*-Butyl-N^6^-Fmoc)-L-Lys-Urea-(Di-*Tert*-Butyl)-L-Glu (**4**)

Compound **3** (1.0 eqv, 1 g) was dissolved in dichloromethane solution and stirred in an ice bath at 0 °C for 5 min. Triphosgene (0.4 eqv, 0.28 g) was then added. The reaction was complete after about 1–2 h, monitored by TLC. Compound **1** (1.0 eqv, 0.612 g) mixed with diisopropylethylamine (4.0 eqv, 1.22 g) were added in an ice bath for 10 min, then transferred to room temperature and stirred until the reaction was completed. Purification by silica gel column chromatography (petroleum ether/ethyl acetate, 4:1) as a colorless transparent viscous solid at room temperature. Yield: 75.3%. ^1^H NMR (400 MHz, CDCl_3_) *δ*: 7.75 (d, *J* = 7.5 Hz, 2H), 7.61 (t, *J* = 6.4 Hz, 2H), 7.39 (t, *J* = 7.4 Hz, 2H), 7.30 (td, *J* = 7.4, 0.9 Hz, 2H), 5.30 (d, *J* = 8.1 Hz, 3H), 4.48–4.28 (m, 4H), 4.21(s, 1H), 3.18 (s, 2H), 2.28 (d, *J* = 6.8 Hz, 2H), 2.04 (s, 1H), 1.91–1.79 (m, 1H), 1.67–1.29(m, 32H). ^13^C NMR (101 MHz, CDCl_3_) *δ*:172.60, 172.49, 172.38, 156.98, 156.65, 144.07, 141.30, 127.63, 127.04, 125.17, 119.92, 82.16, 81.73, 80.52, 66.55, 53.35, 53.05, 47.33, 40.67, 32.69, 31.60, 29.34, 28.33, 28.08, 28.04, 28.01, 22.37. HR-MS(ESI) *m*/*z*: Calcd for C_39_H_55_N_3_O_9_{[M + Na]^+^} 732.38275, found 732.38305.

#### 2.2.5. (*Tert*-Butyl)-L-Lys-Urea-(Di-*Tert*-Butyl)-L-Glu (**5**)

Compound **4** (2 g) was dissolved in a 10% solution of diethyl amine and stirred at room temperature. The mixture was concentrated in vacuo when the reaction was completed. Purification by column chromatography on silica gel (ethyl acetate/methanol, 20:1) provided compound **5** as a yellow sticky solid. Yield: 70%. ^1^H NMR (400 MHz, MeOD) *δ*: 4.02 (ddd, *J* = 16.9, 8.4, 5.1 Hz, 2H), 2.51 (t, *J* = 7.1 Hz, 2H), 2.26–2.02 (m, 2H), 1.88 (dd, *J* = 7.8, 5.9 Hz, 1H), 1.62 (s, 2H), 1.52–1.42 (m, 1H), 1.42–1.22 (m, 31H). ^13^C NMR (101 MHz, MeOD) *δ*: 172.53, 172.33, 172.08, 158.56, 81.38, 81.15, 80.33, 53.41, 52.80, 40.72, 31.99, 31.32, 31.13, 27.63, 27.02, 26.97, 26.94, 22.53. HR-MS(ESI) *m*/*z*: Calcd for C_24_H_45_N_3_O_7_{[M + H]^+^} 488.33295, found 488.33303.

#### 2.2.6. Di-*Tert*-Butyl (((S)-1-(*Tert*-Butoxy)-6-(((4-Nitrophenoxy)Carbonyl)Amino)-1-Oxohexan-2-yl) Carbamoyl)-L-Glutamate (**6**)

Compound **5** (1.0 eqv, 1 g), triethylamine (1.0 eqv, 0.21 g) was dissolved in dichloromethane (20 mL) and then *p*-nitrophenyl chloroformate (1.2 eqv, 0.49 g) was added. The mixture was stirred at room temperature. When the reaction was complete, the mixture concentrated in vacuo. Purification by column chromatography on silica gel (petroleum ether/ethyl acetate, 2:1) provided compound **6** as a colorless transparent viscous solid, yield: 73%. ^1^H NMR (400 MHz, CDCl_3_) *δ*: 8.288.20 (m, 2H), 7.39–7.30 (m, 2H), 6.01 (s, 1H), 5.29 (d, *J* = 8.1 Hz, 1H), 5.20 (s, 1H), 4.37 (qd, *J* = 8.1, 4.8 Hz, 2H), 3.27 (d, *J* = 6.0 Hz,2H), 2.45–2.22 (m, 2H), 2.15–2.02 (m, 1H), 1.95–1.72 (m, 2H), 1.65–1.54 (m, 3H), 1.45 (dd, *J* = 10.8, 7.6 Hz, 29H). ^13^C NMR (101 MHz, CDCl_3_) *δ*: 173.29, 172.37, 172.22, 157.25, 156.33, 153.42, 144.52, 125.03, 121.89, 82.58, 81.80, 80.67, 53.27, 53.01, 41.06, 32.72, 31.54, 28.81, 28.16, 28.05, 28.01, 22.55. HR-MS(ESI) *m*/*z*: Calcd for C_31_H_48_N_4_O_11_{[M + H]^+^} 675.32105, found, 675.32118.

#### 2.2.7. (((S)-1-Carboxy-5-(((4-Nitrophenoxy)Carbonyl)Amino)Pentyl)Carbamoyl)-L-Glutamic Acid (PSMA-1)

The compound **6** (1.0 eqv, 1 g) was dissolved in a dichloromethane solution containing 25% TFA for 16 h. The mixture concentrated in vacuo and purification by column chromatography on silica gel (Methanol/ethyl acetate, 1:1) provided compound PSMA-1 as a colorless, transparent viscous solid, yield: 85%. ^1^H NMR (400 MHz, MeOD) δ: 8.27 (d, *J* = 9.1 Hz, 2H), 7.37 (d, *J* = 9.1 Hz, 2H), 4.33 (ddd, *J* = 13.2, 8.3, 5.0 Hz, 2H), 3.23 (t, *J* = 6.8 Hz, 2H), 2.49–2.37 (m, 2H), 2.23–2.08 (m, 1H), 2.00–1.81 (m, 2H), 1.71 (dd, J = 14.0, 7.3 Hz, 1H), 1.62 (dd, *J* = 11.4, 7.0 Hz, 2H), 1.51 (dd, *J* = 14.9, 7.5 Hz, 2H). ^13^C NMR (101 MHz, MeOD) δ: 175.21, 174.63, 158.74, 156.36, 154.30, 144.70, 124.68, 122.00, 52.68, 52.21, 40.47, 31.65, 29.76, 28.78, 27.55, 22.45. HR-MS(ESI) *m*/*z*: Calcd for C_19_H_24_N_4_O_11_{[M − H]^−^} 483.13697, found 483.13688, ${\left[\alpha \right]}_{D}^{20}$ = 11.3(C = 1, CH_3_OH).

#### 2.2.8. Fe_3_O_4_ SPIONs

FeSO_4_(NH_4_)_2_SO_4_·6H_2_O was dissolved in water (15 mL) for use, NaOH (1 g), oleic acid (10 mL) and ethanol (15 mL) were mixed and stirred to form a homogeneous paste solid, and then the (NH_4_)_2_SO_4_·6H_2_O solution was poured into the mixture and stirred until brown insoluble solids formed. The mixture was then transferred to a hydrothermal reactor (50 mL) with polytetrafluoroethylene liner and allowed to react at 180 °C for 10 h. The reaction kettle was cooled at room temperature. The upper layer solution was removed with a pipette. The lower black solid was pipetted into a 50 mL centrifuge tube. An appropriate amount of ethanol was added, which was centrifuged and then the centrifugate was discarded. After repeating the above operation 3 times, the black substance at the bottom of the centrifuge tube was oleic acid coated Fe_3_O_4_ nano particles, which were finally dispersed in a cyclohexane/chloroform solution and stored at 4 °C.

#### 2.2.9. NH_2_-PEG2000-NH_2_

Polyethylene glycol 2000 (1.0 eqv, 5 g) and triethylamine (2.0 eqv, 0.51 g) were dissolved in dichloromethane (50 mL) solution. After stirring in an ice bath for 10 min, triphosgene (1.0 eqv, 0.75 g) was added. Ethylenediamine (4.0 eqv, 0.6 g) was slowly added after 2 h in the ice bath. The mixture was then transferred to room temperature and stirring continued for 6 h. The mixture was then extracted with dichloromethane and aqueous solution. The organic phase was collected, dried, filtered, and concentrated in vacuo. The crude product was added into 20 mL n-hexane/dichloromethane (5:1) solution and transferred to the refrigerator at −16 °C overnight. Filtered at low temperature, collected, and dried to obtain the compound NH_2_-DPA-PEG (2000)-NH_2_ as a white solid, yield: 50%. The main characteristic peaks of the compound were as follows: 1H NMR (400 MHz, CDCl_3_), 4.21 (s, 4H), 3.64 (s, 227H), 3.28 (s, 4H).

#### 2.2.10. DPA-PEG-NH_2_

A solution of NH_2_-PEG (2000)-NH_2_ (1.0 eqv, 1 g) in dichloromethane (20 mL) solution was stirred for 5 min in an ice bath. Then triphosgene (0.4 eqv, 55 mg) was added. A solution of dopamine hydrochloride (2.0 eqv, 175 mg) in pyridine (20 mL) solution was added after 1 h. After stirring at room temperature for 6 h, dichloromethane (50 mL) solution was added, and extracted with water (30 mL × 3). The organic solution was dried (Na_2_SO_4_), filtered, and concentrated in vacuo. The crude material was dissolved in *n*-hexane/dichloromethane (5:1, 20 mL) and stored at −16 °C. The precipitated solid was collected and dried. Compound DPA-PEG (2000)-NH_2_ was then obtained. The main characteristic peaks of the compounds were as follows: ^1^H NMR (400 MHz, CDCl_3_) *δ* 6.84 to 6.75 (m, 1H), 6.73 (s, 1H), 6.55 (d, *J* = 8.0 Hz, 1H), 4.20 (d, *J* = 4.6 Hz, 4H), 3.63 (d, *J* = 9.6 Hz, 200H), 3.31–3.20 (m, 4H). 

#### 2.2.11. Fe_3_O_4_@DPA-PEG-NH_2_

A total of 600 mg of DPA-PEG(2000)-NH_2_, 100 ul of TEA and 100 mg of nano-Fe_3_O_4_ particles were dissolved. The reaction was vigorously stirred in 20 mL of dichloromethane solution for 6 h. After the reaction, part of the solvent was evaporated under reduced pressure at room temperature, and the remaining concentrated solution was sucked into a centrifuge tube, and an appropriate amount of cyclohexane was added for centrifugation. The supernatant was discarded, and the operation was repeated twice. The black solid obtained by centrifugation was placed at 35 °C for 30 min under reduced pressure to obtain target product Fe_3_O_4_@DPA-PEG-NH_2_ 216 mg. The product was dispersed in 3 mL of PBS solution.

#### 2.2.12. Fe_3_O_4_@DPA-PEG-PSMA-1

The Fe_3_O_4_@DPA-PEG-NH_2_ obtained in the previous step and dissolved in chloroform solution (30 mL) was transferred into a 100 mL reaction flask. A solution of PSMA-1(1.0 eqv verses DPA-PEG(2000)-NH_2_, 102 mg) and DIEA (100 μL) in methanol (10 mL) solution was added. Then, the reaction was stirred for 6 h and then concentrated in vacuo. The concentrated solution was transferred to a centrifuge tube, and an appropriate amount of cyclohexane/methanol solution (5:1) was added for centrifugation. The supernatant was collected. After repeated operations twice, the black solid obtained was evaporated under reduced pressure at 35 °C for 30 min. The desired product target product Fe_3_O_4_@DPA-PEG-PSMA-1 obtained was 148 mg and then dispersed in PBS (3 mL) solution, and DIEA (10 μL) was added to stabilize the probe in the aqueous solution. The supernatant was combined and then purified by column chromatography on silica gel (methanol/ethyl acetate, 1:1) to afford remaining unlabeled PSMA-1 was 89 mg.

### 2.3. Characterization

^1^H and ^13^C NMR spectra were recorded using TMS as the internal standard in CDCl_3_ with a Bruker BioSpin GmbH spectrometer at 400 MHz and 101 MHz, respectively, with chemical shifts (δ) in ppm. Low resolution ESI mass spectra were determined on a Bruker Daltonics Esquire 3000 Plus mass spectrometer. Ultra-high-resolution mass spectrum was performed on Q Exactive Plus Orbitrap LC-MS/MS System (Thermo Fisher Scientific, Waltham, MA, USA). FTIR spectroscopy was recorded with Thermo Fisher Nicolet 6700 (Thermo Fisher Scientific, USA). X-ray diffraction was performed on X-ray Single Cristal Diffractometer (Bruker D8 VENTURE, Billerica, MA, USA).

The morphology of Fe_3_O_4_, Fe_3_O_4_@DPA-PEG-NH_2_ and Fe_3_O_4_@DPA-PEG-PSMA-1 was photographed with TEM (SU8220, Hitachi, Japan). A particle size analyzer (Brookhaven BI-200SM, Brookhaven, GA, USA) was used to detect the hydrodynamic size distribution. The zeta potential of SPIONs dissolved in PBS or FBS were measured by Malvern Zetasizer Nano ZS (Malvern, Worcestershire, UK). The magnetic properties were evaluated by a vibrating sample magnetometer (Quantum Design VersaLab, San Diego, CA, USA). The iron concentration was examined by the 1, 10-phenanthroline spectrophotometric method on a spectrophotometer (UV-3600, Shimadzu, Japan). The T_2_-weighted MRI imaging of Fe_3_O_4_@DPA-PEG-NH_2_ was carried out on a 1.0T M3 Compact MRI (Aspect Imaging Ltd., Shoham, Israel).

### 2.4. PSMA Biding Assay

#### 2.4.1. Cytotoxicity Assay (MTT & LDH)

MTT assay: LNcaP and HaCaT cells were added to 96-well plates with 5000 cells per well. The complete medium was added and cultured overnight. Tested drugs were added in wells with final concentrations of 0 μg/mL, 7.5 μg/mL, 15 μg/mL, 30 μg/mL, 60 μg/mL, and 120 μg/mL, respectively. And five replicate wells were set for each concentration. After culturing the cells for 48 h, the medium was removed, and the cells were washed twice with PBS solution. A solution of 100 μL MTT (Aladdin, Shanghai, China) was added to each well in the dark and transferred to a cell incubator for 3–4 h of incubation. After incubation, the MTT solution in the well plate was discarded, 100 μL/well DMSO solution was added and shaken for 20 min, then the absorbance (OD) value at 570 nm was measured with a microplate reader (Bio-Tek ELx800 plate reader, Bio-Tek, Winooski, VT, USA). The raw values for all the assays were normalized by the controls and expressed as percent viability.

LDH assay: Cytotoxicity LDH Assay Kit-WST (Dojindo, Beijing, China) was used to evaluate cytotoxicity. LNcaP and HaCaT cells were added to 96-well plates with 5000 cells per well. The complete medium was added and cultured overnight. Tested drugs were added in wells with final concentrations of 0 μg/mL, 7.5 μg/mL, 15 μg/mL, 30 μg/mL, 60 μg/mL, and 120 μg/mL, respectively. And five replicate wells were set for each concentration. After incubation for 24 h, 15 μL of lysis buffer was added to high control. After incubation for 30 min, 100 μL cellular supernatants of each well was transferred to another 96-well plate and 100 μL working solution was added to each well. The plate was incubated in the dark for 30 min at room temperature. Next, 50 µL of the stop solution was added into each well. The absorbance at 490 nm was measured using a microplate reader (Bio-Tek ELx800 plate reader, Bio-Tek, Winooski, VT, USA). LDA released was normalized by High Control. Cytotoxicity was calculated by a following equation: Cytotoxicity (%) = 100 × (Test Drug − Low Control)/(High Control − Low Control).

#### 2.4.2. PSMA Molecular Binding Assay

The PSMA inhibition assay was performed using a fluorescence-based assay as previously described procedures [28,29,30] with minor modification. Lysates of LNCaP cell extracts (40 µL, containing 10 µg total protein, 50 mM TrisCl, 1 mM CoCl_2_, pH 7.4 at 37 °C) were added with samples of different concentrations (20 µL) and then incubated in 4 µM NAAG (20 µL) for 120 min. The amount of glutamate released from NAAG hydrolysis was determined with a Glutamate Assay Kit (Abnova, Taipei, Taiwan), as described in the standard protocol. Optical density (OD_0_) for time “zero” was read at 565 nm (520–600 nm) and OD_30_ after a 30-min incubation at room temperature. For all samples, dose-response curves were plotted and IC_50_ values were calculated by nonlinear regression using GraphPad Prism version 8.0.2 for Windows (GraphPad Software, San Diego, CA, USA). Enzyme inhibitory constants (*K*_i_ values) were calculated from the respective IC_50_ and *K*_m_ values using the Cheng-Prusoff conversion.

#### 2.4.3. Cell Binding Assay

LNCaP cells (PSMA positive), PC3 cells (PSMA negative), liver hepatocellular carcinoma HepG2 cells and human immortalized keratinocytes HaCaT cells were procured from National Infrastructure of Cell Line Resource (NICLR, Beijing, China). Cells were cultivated in a 37 °C, 5% CO_2_ incubator using RPMI-1640 media (Gibco) supplemented with 10% fetal bovine serum (Invitrogen, Waltham, MA, USA), 100 U/mL penicillin G, and 0.1 mg/mL of streptomycin (Invitrogen).

Prussian blue staining was adopted to develop the color of SPIONs. LNcaP, PC3 and HepG2 were seeded in three 6-well plates, respectively, with a cell density of 2 × 10^5^ cells/mL. Each kind of cell was incubated with empty vehicle, Fe_3_O_4_@DPA-PEG-NH_2_ or Fe_3_O_4_@DPA-PEG- PSMA-1 (Fe, 20 μg/mL) for 4 h. After incubation, PBS was used to rinse cells three times to remove free SPIONs. Cells were then fixed with 4% paraformaldehyde for 10 min, rinsed with distilled water twice, and then treated with Perls reaction liquid for 30 min. After rinsing and counterstaining, the blue granules inside cells, which are supposed to be cellular uptake of SPIONs, were observed and photographed under a microscope (BDS-DM500, Chongqing OP Tec Instrument Co., Ltd., Chongqing, China) at 200× magnification.

#### 2.4.4. Western Blot of PSMA

LNCaP cells, PC3 cells and HepG2 cells were homogenized in 2 mL phosphate-buffer saline (PBS, pH 7.4) and centrifuged at 12,000 rpm for 15 min/4 °C to remove cell debris. Equal amounts of protein of the supernatant were separated by SDS-PAGE on 8–12% Bis-Tris gels, transferred to nitrocellulose membranes, and incubated overnight at 4 °C with PSMA antibody (Abcam EPR6253, Cambridge, UK). Subsequently, the membranes were incubated for 2 h with the corresponding secondary antibody. The immunodetection was done using an enhanced chemiluminescence detection kit (MultiSciences Biotech, Hangzhou, China). The bands density was quantified using the Quantity One analysis software (Bio-Rad, Hercules, CA, USA) via calculating the average optical density in each field.

### 2.5. In Vivo MRI

#### 2.5.1. Animal Model

All animal experimental procedures were performed in accordance with the National Institutes of Health guide for the care and use of laboratory animals (NIH Publications No. 8023, revised 1978) and were approval by Animal Care and Use Committees of Experimental Animal Center of Guangdong University of Technology. Nude mice (male BALB/c mice, 5–6 weeks) were obtained from SPF Biotechnology Co., Ltd. (SCXK 2016-0002, Beijing, China). All mice were hosted in a pathogen-free animal room with controlled temperature at 23 ± 2 °C, 12/12 h light/dark cycles, and provided with standard laboratory diet and water. In order to establish a PCa subcutaneous xenograft model, each mouse was injected with 3 × 10^6^ LNCaP cells/200 μL RPMI-1640 media blended with 100 μL Corning Matrigel (BD Biocoat, New York, NY, USA). After injection, tumor size on mice was measured 3 times a week (length, width, and height). The tumors were considered as ellipsoids and their volume was calculated using the formula for ellipsoid, i.e., 0.52 × height × length × width. When the tumor volume reached 0.5 mL, the mice were set for MRI studies. The animal experiment in the study was performed in accordance with ethical standards approved by Animal Care and Use Committees of Experimental Animal Center of Guangdong University of Technology.

#### 2.5.2. In Vivo MRI

Model mice implanted with LNCaP cells were anesthetized with amobarbital (0.6%, 1.0 mL/100 g body weight) via intraperitoneal injection. All mice were divided into 2 groups. Each group of mice were injected with each dose of Fe_3_ O_4_@DPA-PEG-PSMA-1 or Fe_3_O_4_@DPA-PEG-NH_2_ (Fe, 20 mg/kg) intravenously via the tail vein. All images of in vivo MRI were taken before and 1, 3, 6, and 24 h after injection using a 1.0T M3 Compact MRI (Aspect Imaging Ltd., Shoham, Israel). The imaging parameters were set as follows: repetition time (TR) = 3250 ms, echo time (TE) = 63.8 ms, field of view = 35 × 35 mm, matrix = 384 × 384, slice thickness = 2 mm, number of slices = 11, acquisition time = 130 s.

#### 2.5.3. Histological Analysis

Mice were euthanized with CO_2_ after the MRI test. Harvested tumor, liver and spleen tissues were fixed with 4% paraformaldehyde, embedded and sectioned at a thickness of 5 μm. The sections were then incubated in 3% H_2_O_2_/MeOH for 30 min for immunohistochemical staining. In Prussian blue staining, the sections were rinsed in distilled water and then incubated in Perls reaction liquid for 30 min. After rinsing and counterstaining, microscopic images (×400) for the deposition of blue stain were captured by an inverted fluorescence microscope (BDS-DM500, Chongqing OP Tec Instrument Co., Ltd., Chongqing, China).

### 2.6. Statistical Analysis

All data were reported as mean ± SD. Statistics were analyzed with Prism GraphPad (v7.0.4, GraphPad Software, San Diego, CA, USA) using a one-way ANOVA followed by the LSD post hoc test for multiple comparisons. *p* values less than 0.05 (*p* < 0.05) were considered statistically significant.

## 3. Results and Discussion

### 3.1. Synthesis and Characterization

#### 3.1.1. Design and Synthesis of MRI Probe

**Figure 1:**
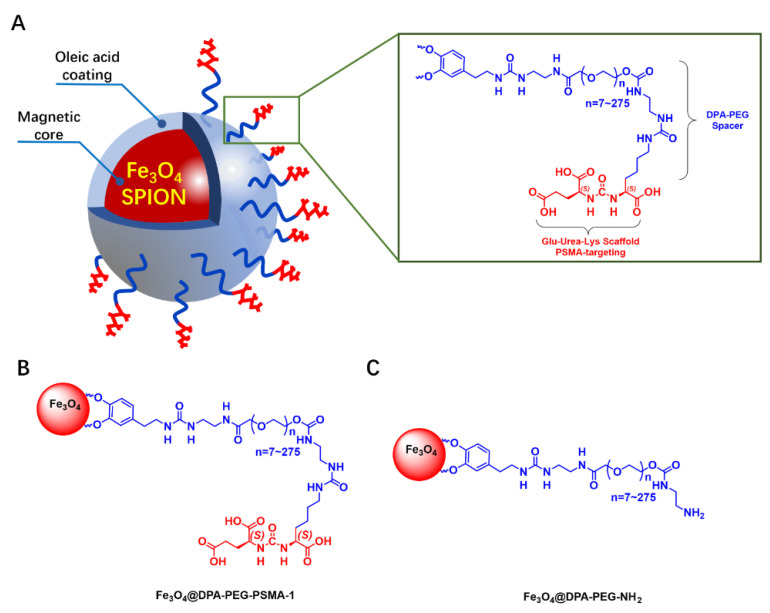
Design of the PSMA MRI probe and the negative control probe. (**A**) a schematic illustration of Glu-Urea-Lys scaffold functionalized SPION probe for MRI of PCa. (**B**,**C**) SPIONs labelled with/without PSMA-targeting Glu-Urea-Lys scaffold (PSMA-1).

In the design of our PSMA MRI molecular imaging probe for PCa, a Glu-Urea-Lys motif was adopted (Figure 1). The Glu-Urea-Lys scaffold has been extensively validated in many kinds of PSMA probes exemplified by the PET tracer for PSMA ([^68^Ga]-PSMA-HBED-CC) in clinical use for its favorable specificity, affinity and safety [31]. Compared with nanoparticles labelled with antibodies, probes labelled with PSMA-targeting small molecules has many advantages, including stability, easy conjugation, and potentially low cost [32]. The hydrophilic Fe_3_O_4_ superparamagnetic nanoparticles were prepared and attached to the Glu-Urea-Lys pharmacophore (PSMA-1) with biologically compatible polyethylene glycol (PEG, Figure 1) as the pharmacokinetic tuner. Another probe (Fe_3_O_4_@DPA-PEG-NH_2_) without PSMA-specific ligand (PSMA-1) was also prepared for negative control in our study.

**Scheme 1:**
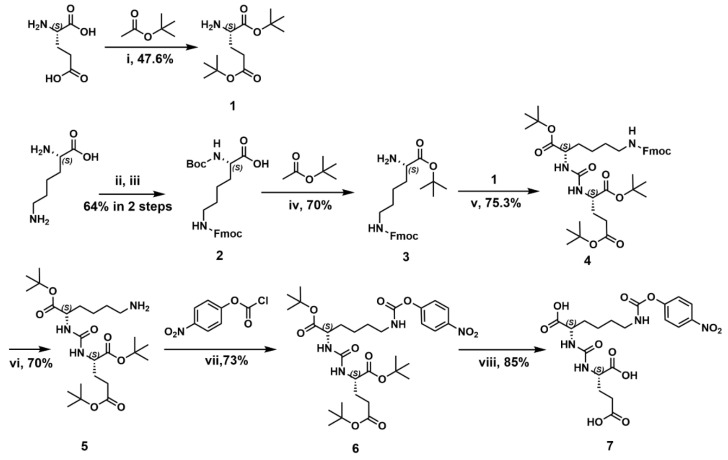
The synthesis of PSMA-1. Reagents and conditions: (i) 70% HClO_4_, 47.6%; (ii) Fmoc-OSU, CuSO_4_, NaHCO_3_; (iii) Na_2_CO_3_, 8-hydroxyquinoline, Boc_2_O, 64% in 2 steps; (iv) 70% HClO_4_, 70%; (v) triphosgene, DIEA, 75.3%; (vi) DEA, 70%; (vii) DEA, CH_2_Cl_2_, 73%; (viii) 25%TFA/CH_2_Cl_2_, 85%.

The construction of PSMA-1 was, according to the schematic illustration in Scheme 1, based on previously reported research with modification [33]. Carbonyl protected *L*-glutamine (**1**) and *L*-lysine (**3**) were key intermediates. Since the free *ε* amino group of lysine was preserved for derivatization with PEG, the *α* and *ε* amine amino groups of lysine were protected with a *tert*-butoxycarbonyl (Boc) and a fluorenylmethoxycarbony (Fmoc) groups, respectively. An effort was made to optimize the selective protection of *L*-lysine. Copper (II) ion was employed to induce chelation of the *α* amino group and the carboxyl group, which allowed the free *ε* amino group protection first. Fmoc group was much more convenient to be deprotected (with diethyl amine) compared to Boc protection (with Pt/C+H_2_) [23,33]. Meanwhile, we developed the Boc deprotection of *α* amino group and protection of *α* carbonyl group in signal one step with good yield (70%). Triphosgene was used to connect protected glutamine and lysine, forming a urea scaffold. Our synthetic route provided a convenient approach for the preparation of PSMA-1 with acceptable total yield (18%). The chemical structure of PSMA-1 and intermediates were confirmed by nuclear magnetic resonance (NMR) and high-resolution mass spectrometry (HRMS) in Supplementary Materials. The scheme provided a feasible synthetic route for the construction of PSMA-1 with cheap raw materials under relatively ambient conditions. With commercially available protected amino acids, e.g., compound **1** and **3**, the total synthetic route can be simplified to four steps, with dramatically higher total yield.

**Figure 2:**
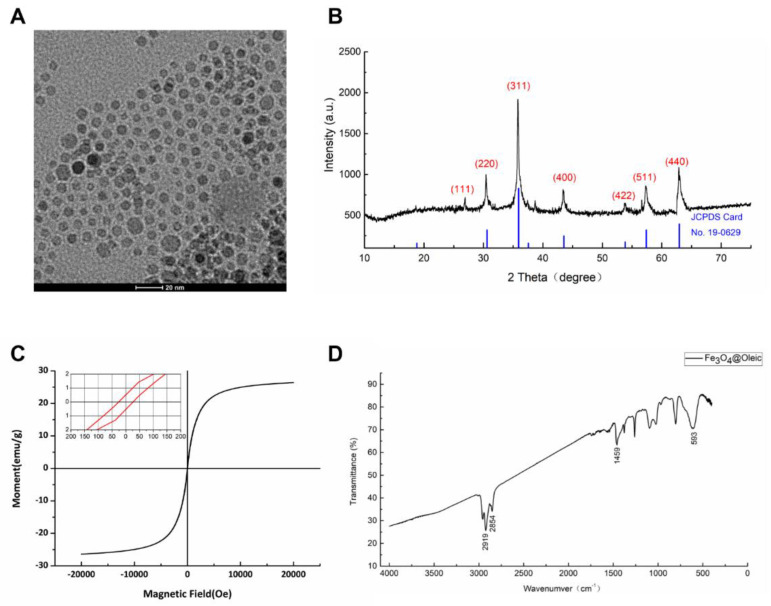
Characterization of SPIONs (F_3_O_4_ nanoparticles). (**A**) Representative transmission electron microscopic image of F_3_O_4_ nanoparticles. The scale bar on the TEM image equals 20 nm. (**B**) X-ray diffraction patterns of F_3_O_4_ nanoparticles. (**C**) VSM curve of F_3_O_4_ nanoparticles. (**D**) FTIR spectrum of F_3_O_4_ nanoparticles.

In order to obtain stable SPIONs, a hydrothermal method was carried out to prepare the oleic acid (OA) coated iron oxide nanoparticles within an oleic acid/alcohol/water system [34]. The transmission electron microscopy (TEM) image in Figure 2A showed spherically shaped, monodispersed partials with an average size of 8–10 nm in diameter.

According to the figure of X-ray powder diffraction (XRD, Figure 2B), sharp and intense diffraction peaks were observed, indicating its highly crystalline nature. Six typical peaks for SPIONs (2θ = 30.37°, 35.75°, 43.39°, 53.82°, 57.28° and 62.79°) corresponding to the *hkl* values ((220), (311), (400), (422), (511), and (440)) respectively were consistent with the characteristics of face-centered cube magnetite Fe_3_O_4_ (JCPDS card No. 19-0629) [35].

The magnetic behavior of SPIONs was measured with a vibrating sample magnetometer. In an applied magnetic field, the magnetization of nanoparticles rose along with increasing applied magnetic field strength and reached a saturation magnetization (Ms) value of 27 emu/g (27 Am^2^/kg sample weight after cryodesiccation). Near-zero hysteresis demonstrated by remanent magnetization 0.5 Am^2^/kg (Fe) and coercivity 25 Oe (1.99×10^3^ A/m) could be observed in the magnetization curve, indicating the superparamagnetic property of the iron nanoparticles (Figure 2C).

The Fourier Transform Infrared (FTIR) analysis of Fe_3_O_4_ oleic nanoparticles exhibited strong peaks of CH_2_ bands at 2919 (*ν*_a_ C-H) and 2854 (*ν*_s_ C-H) (Figure 2D), which were known to be the characteristic bands of CH_2_ groups presented in oleic acid [36]. The band at 580 cm^−1^ could be referred to the vibration of the Fe-O bonds in the crystalline lattice of Fe_3_O_4_. The band at 1459 cm^−1^ was intended to be attributed to the stretch vibration band of C=O [37]. Based on the FTIR spectra, oleic acid was believed to coat the surface of the iron nanoparticles.

Now that the prepared magnetic cores were validated as superparamagnetic Fe_3_O_4_ nanoparticles, we moved on to conjugate the PSMA specific ligand (PSMA-1) to the SPIONs.

Modified polyethylene glycol 2000 (PEG 2000) was utilized as a linker to connect SPIONs with PSMA-specific ligand (PSMA-1). It was previously demonstrated that PEG 2000 coating SPIONs provided moderate stability and magnetic contrast and showed slight cytotoxicity and non-specific cellular uptake [38]. As shown in Figure 1, PEG 2000 was functionalized with dopamine at one end to anchor to the SPION, and with ethane diamine at the other end for condensation with the free amine of PSMA-1. SPIONs equipped with the synthetic ligand PSMA-1 were prepared (Fe_3_O_4_@DPA-PEG-PSMA-1) using the method of ligand exchange. According to the labelling process, the loading capacity of PSMA-1 on SPIONs was determined as 8.9 wt.% based on the final product and the unlabeled PSMA-1 remained in the supernatant. Final SPIONs (Fe_3_O_4_@DPA-PEG-PSMA-1), along with PEG-labelled SPIONs without PSMA-targeting ligand as the negative control (Fe_3_O_4_@DPA-PEG-NH_2_), were both engaged in characterization and function assays for comparison.

#### 3.1.2. Characterization of SPIONs

**Figure 3:**
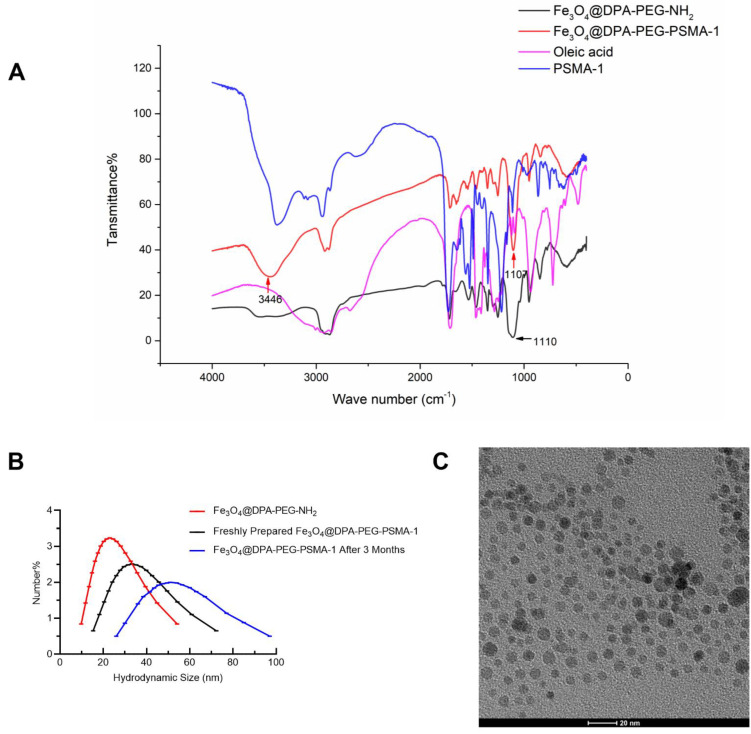
Characterization of Fe_3_O_4_@DPA-PEG-NH_2_ and Fe_3_O_4_@DPA-PEG-PSMA-1. (**A**) FTIR spectra of Fe_3_O_4_@DPA-PEG-NH_2_ (Black), Fe_3_O_4_@DPA-PEG-PSMA-1 (Red) PSMA-1 (Blue) and oleid acid (Magenta). (**B**) Comparison of hydration particle size distribution of Fe_3_O_4_@DPA-PEG-NH_2_ (Red), freshly prepared Fe_3_O_4_@DPA-PEG-PSMA-1 (Black), and 3 months post preparation (Blue). (**C**) Representative transmission electron microscopic image of Fe_3_O_4_@DPA-PEG-PSMA-1. The scale bar in the TEM image equals 20 nm.

According to the FTIR spectra (Figure 3A), the broad band at 3446 cm^−1^ was the main difference between spectra of Fe_3_O_4_@DPA-PEG-PSMA-1 and Fe_3_O_4_@DPA-PEG-NH_2_. This absorption could be ascribed to the vibrations of O-H of the carboxylic acid groups in PSMA ligand, like the absorption at 3388 cm^−1^ of PSMA-1. The vibrations of NH_2_ exemplified as the broad band at approximately 3200–3500 cm^−1^ could be conferred as another characteristic difference between Fe_3_O_4_@DPA-PEG-NH_2_ and Fe_3_O_4_@DPA-PEG-PSMA-1. Typical bands at 1107 cm^−1^ of Fe_3_O_4_@DPA-PEG-PSMA-1 and 1110 cm^−1^ of Fe_3_O_4_@DPA-PEG-NH_2_ could be observed, which it was possible to assign to the asymmetric stretch vibration of C-O-C bonds of PEG fragment. Other sharp bands between 1200–1700 cm^−1^ of PSMA-1 were preserved in Fe_3_O_4_@DPA-PEG-PSMA-1, though these were not of great strength. Based on the FTIR spectra, the desired SPIONs conjunct with PEG linker and PSMA ligands were confirmed preliminarily.

The size distribution of nanoparticles is one of the crucial factors of stability, tissue permeability, bio-distribution, and cellular internalization of drug delivery dynamic of nano drugs [39]. In our work, both dynamic lights scattering (DLS), and transmission electron microscopy (TEM) methods were employed for the particle sizing of both Fe_3_O_4_@DPA-PEG-NH_2_ and Fe_3_O_4_@DPA-PEG-PSMA-1. Oleic acid coated Fe_3_O_4_ became well-dispersed in water, as well as in phosphate-buffered saline (PBS), after being incorporated with PEG chains, without changing particle sizing. In Figure 3B, it showed a narrow distribution of the hydrodynamic diameter of Fe_3_O_4_@DPA-PEG-NH_2_ 25.4 ± 9.0 nm (mean ± SD, red curve) and Fe_3_O_4_@DPA-PEG-PSMA-1 35.1 ± 11.6 nm (black curve) in water, respectively. As shown in TEM images (Figure 3C), particles presented uniform and sphere-like shape morphology with an approximate diameter of 8–12 nm. Uniform size particles offer equal probability of magnetic capture of drug-loaded nanoparticles and are characterized by similar drug content. DLS is expected to measure the hydrodynamic radius rather than the actual size of the nanoparticles. Considering dispersing solvent molecules adherent to the surface of nanoparticles in DLS measurement [40], the size discrepancy of SPIONs detected by DLS or TEM methods was consistent in principle. The size of SPIONs is critical for the cellular uptake and their inside-cell fate. Exemplified by the research of gold nanoparticles (AuNP), smaller AuNP resulted in better in vivo radio-sensibility and a lower level of liver uptake, and may lower off-target elemental toxicity [41]. It is suggested that the nanoparticles with a radius under 30 nm are quickly up-taken by pinocytosis [42]. Arguably, it could be the smallest PSMA-targeting SPIONs in diameter to our knowledge [20,22,27], which may contribute to better tissue permeability and in vivo MR molecular imaging.

Hydrophilic coating can significantly decrease SPION aggregation through electrostatic interactions or steric hindrance, which could eventually contribute to the stability of SPION. Zeta potential, a typical indicator of surface charge of nanoparticles in a colloidal suspension, is generally used to define the stability and aggregation tendency of nanoparticles. The zeta potential of SPIONs was measured at pH 7. As shown in Table 1, the zeta potential of oleic acid-coated F_3_O_4_ nanoparticles, Fe_3_O_4_@DPA-PEG-NH_2_ and Fe_3_O_4_@DPA-PEG-PSMA-1 in PBS (pH7.4) were −25.2, 18.8, and −22.4 mV, respectively. Deprotonation of -OH group of PEG or carboxylic acid was the supposed cause the formation of negative charges on nanoparticle surface, resulting in the negative zeta potential at near-neutral pH. When suspended in FBS which was set to more closely mimic the physiological conditions in vivo, zeta potential of Fe_3_O_4_@DPA-PEG-PSMA-1 was relatively high compared to that in PBS, indicating that binding or adsorption of complicated composition such as proteins on the surface of nanoparticles may have a slightly negative impact on the in serum, which could potentially alter the aggregation state and cellular response. More importantly, modification with PEG was generally recognized to reduce protein adsorption and then decrease non-specific cellular uptake [43]. It was concluded that Fe_3_O_4_@DPA-PEG-PSMA-1 presented overall modest stability in vitro, presumably also metabolic stability when injected in vivo. Besides, the physicochemical stability was confirmed that the hydrodynamic diameter of SPIONs (Fe_3_O_4_@DPA-PEG-PSMA-1) shifted from 35.1 nm to 52.2 nm (Figure 3B, blue curve), despite being dispersed in PBS for three months. The increase in the hydrodynamic radius was possibly due to the decrease in stability generated by electrostatic interactions or steric hindrance corrupted by constant Van der Waals forces and Brownian movement. 

### 3.2. PSMA Binding Assay

#### 3.2.1. Molecular Binding Assay

The concentration of Fe_3_O_4_@DPA-PEG-PSMA-1 suspension was determined from a standard curve of iron standard solution (160 μg/mL) via the Prussian blue staining method. The content of iron in Fe_3_O_4_@DPA-PEG-PSMA-1 PBS solution which was used in subsequent in vitro or in vivo biological assays was 3.0 mg/mL.

We determined the in vitro PSMA inhibitory activity of PSMA-1, Fe_3_O_4_@DPA-PEG-NH_2_ and Fe_3_O_4_@DPA-PEG-PSMA-1 using a fluorescence-based N-Acetylated alpha-linked acidic dipeptidase (NAALADase) inhibition assay, as in previously reported methods [28,29,44]. The NAALADase (also referred as PSMA) was prepared from LNCaP cell lysates. The percentage of glutamate, the hydrolytic product of the natural PSMA substrate N-acetylaspartylglutamate (NAAG) was measured by a glutamic acid kit. *K*_i_ values of the three samples were calculated according to linear regression of logarithmic concentration and effect. As shown in Table 2, the *K*_i_ value of PSMA-1 was 2.4 nM, which was within the same order of magnitude as some of the PSMA monoclonal antibodies (3/A12 mAb 2 nM binding affinity to the extracellular domain of PSMA in LNCap cells) [45]. The modification of the protecting group of *ε* amine of lysine resulted in a slight compromise of the inhibitory activity [28]. During the preparation of the manuscript, a novel series of (S)-3-(Carboxyformamido)-2-(3-(carboxymethyl)ureido)propanoic acids were discovered as more potent PSMA ligands with *K*_i_ values up to 80 pM [46]. However, it is still a long way to examining the metabolism and safety issues of the new scaffold as a matured ligand for probe design. While the SPIONs (Fe_3_O_4_@DPA-PEG-NH_2_) were found to have no obvious inhibitory activity against PSMA, the efficacy of SPIONs labeled with PSMA-1 was calculated as 0.38 μg(Fe)/mL after conversion, since the concentrations of Fe_3_O_4_@DPA-PEG-PSMA-1 were quantified with Prussian blue staining of ferriferous oxide. Also, for this reason, it brought us challenges for directly comparing binding selectivity and the affinity of our probes with other previously reported antibody labelled SPIONs. The binding affinity of Fe_3_O_4_@DPA-PEG-PSMA-1 to PSMA manifested by inhibitory activity showed its promising binding potential with PSMA expressed on PCa cell surface.

#### 3.2.2. Cell Binding Assay

**Figure 4:**
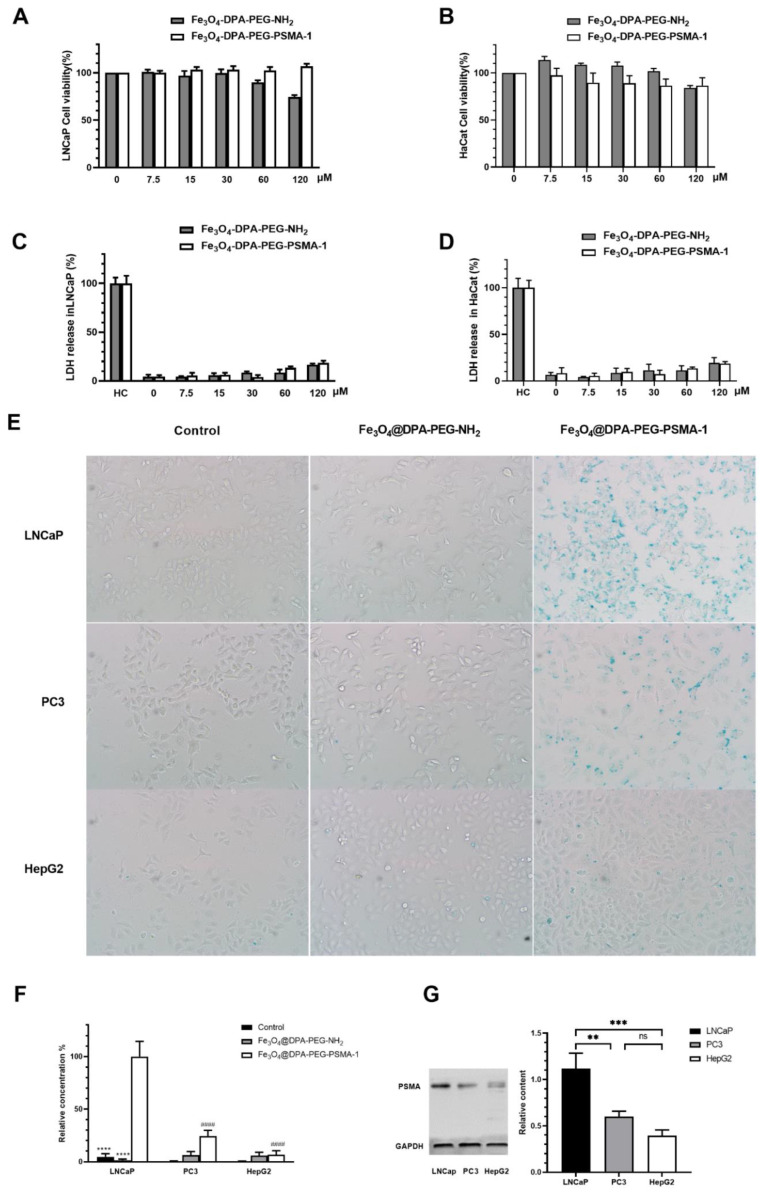
Cell viability of LNCaP (**A**) and HaCat (**B**) by the MTT assay after incubation with different concentration (Fe, μg/mL) of Fe_3_O_4_@DPA-PEG-PSMA-1 and Fe_3_O_4_@DPA-PEG-PSMA-1 for 48 h. Lactate dehydrogenase (LDH) released to cellular supernatant by LNCap cells (**C**) and HaCat (**D**) after 24 h incubation with Fe_3_O_4_@DPA-PEG-PSMA-1 and Fe_3_O_4_@DPA-PEG-PSMA-1. Values were normolized with High Control (HC) containing total LDH. (**E**) In vitro selective binding assay of Fe_3_O_4_@DPA-PEG-PSMA-1. Incubation of 20 μg(Fe)/mL Fe_3_O_4_@DPA-PEG-PSMA-1 in LNCaP (PSMA positive, top row), PC3 (PSMA low expression, middle row) and HepG2 (PSMA negative, bottom row) cells for 4 h, with Fe_3_O_4_@DPA-PEG-NH_2_ (middle column) as negative control and PBS as empty vehicle. Prussian blue was utilized for staining. (**F**) Semi-quantification of relative concentration of iron staining in LNCaP, PC3, and HepG2 cells. (**G**) Western blot bands of PSMA expression and semi-quantification of relative content of PSMA in three cell lines. Data were presented as means ± SD. Compared with Fe_3_O_4_@DPA-PEG-PSMA-1 in tumor tissue, **** *p*< 0.0001, *** *p* < 0.001, ** *p* < 0.01, ns not significant. Compared with Fe_3_O_4_@DPA-PEG-PSMA-1 in LNCap group, ^####^ *p* < 0.0001.

The specific binding affinity of Fe_3_O_4_@DPA-PEG-PSMA-1 was validated using LNCaP cells, PC3 cells and non-PCa HepG2 cells, with Fe_3_O_4_@DPA-PEG-NH_2_ as the negative control. Since free radicals possibly generated by iron oxide nanoparticles themselves [47] or coating material oleic acid [48] may cause cytotoxicity, prior to this assay, the MTT assay showed that the SPIONs were seemingly safe, demonstrated by high cell viability of LNCaP and HaCat cells even at high concentration of 120 μg (Fe)/mL (Figure 4A,B). The lactate dehydrogenase (LDH) assay showed no significant release of LDH from LNCaP and HaCat cells after exposure to various concentrations of Fe_3_O_4_@DPA-PEG-PSMA-1 and Fe_3_O_4_@DPA-PEG-PSMA-1 for 24 h as shown in Figure 4C,D.

In the selective binding assay, LNCaP, PC3 and HepG2 cells were treated with Fe_3_O_4_@DPA-PEG-NH_2_ and Fe_3_O_4_@DPA-PEG-PSMA-1, respectively, at a concentration of 20 μg(Fe)/mL for 4 h. After removing unbinding probes by washing, Prussian blue staining assay was used to stain ferriferous oxide. It could be clearly observed that significantly greater iron, which was stained in blue, adhered to LNCaP cells (PSMA expression positive) treated with Fe_3_O_4_@DPA-PEG-PSMA-1 than to non-targeted Fe_3_O_4_@DPA-PEG-NH_2_ (Figure 4E,F). Blue staining in PC3 cells was much less than in LNCaP cells, while barely any cell was stained in HepG2 cells. It was generally acknowledged that low level of PSMA was expressed on the surface of the PC3 cells [49], and HepG2 cells, immortalized human liver carcinoma cells, were supposed to be PSMA negative. Semi-quantification of Western blot bands of PSMA expressed in the three cell lines also demonstrated that LNCaP cells were PSMA positive and its PSMA expression level was significantly higher than that of PC3 and HepG2 cells (Figure 4G). Taking these into account, labelling of SPIONs with PSMA-targeting ligands facilitated their binding with the LNCaP cells. It should also be noted that the blue stains appeared both on the surface and inside of LNCaP cells, possibly due to the probe taken up by cells via pinocytosis. These results indicated that, similar to PSMA antibody conjugation [50], SPIONs labelled with PSMA-targeting ligands could also specifically bind with PSMA positive PCa cells and be selectively internalized into the cells.

### 3.3. MRI

#### 3.3.1. In Vivo MRI

**Figure 5:**
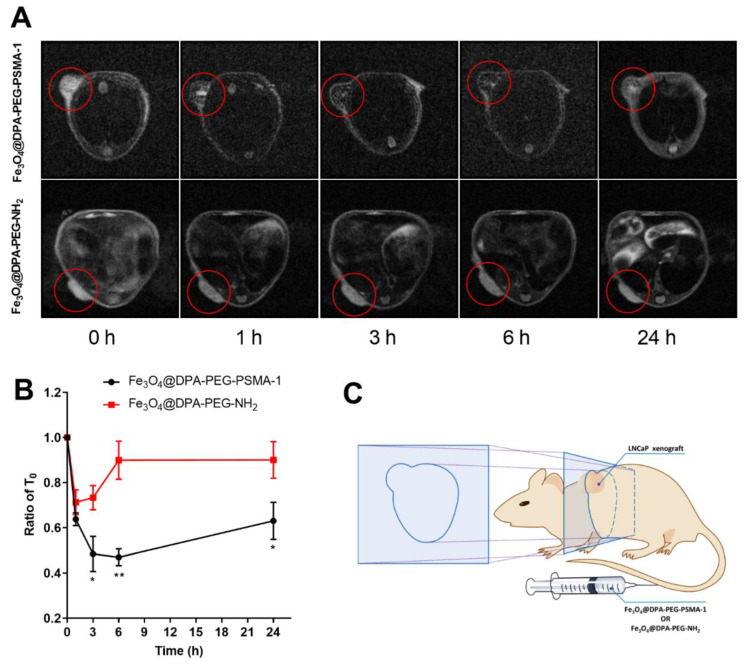
(**A**) In vivo MRI of Fe_3_O_4_@DPA-PEG-PSMA-1 in PCa subcutaneous xenograft model mice in cross section. T_2_ contrasted MR images of mice xenografted with LNCaP cells before and after tail vein injection of Fe_3_O_4_@DPA-PEG-PSMA-1 or Fe_3_O_4_@DPA-PEG-NH_2_ (Fe, 20 mg/kg). (**B**) Time-dependent curve of concentrations of the two probes in tumor area. Data are presented as means ± SD normalized by the intensity at T_0_. Compared with Fe_3_O_4_@DPA-PEG-NH_2_ at each time point, * *p* < 0.05 ** *p* < 0.001. N = 3 per group. (**C**) Schematic illustration of LNCaP xenograft mouse model and the cross section for imaging.

Iron oxide nanoparticles are extensively used in MRI as a T_2_ contrast agent [20,50,51]. Preliminarily, Fe_3_O_4_@DPA-PEG-PSMA-1 was validated as a T_2_ weighted contrast in water in the magnetic field, as the darkening effect increased along with Fe_3_O_4_ concentrations (Supplementary Figure S1). To date, extensive SPIONs have been developed to detect cancers either by conjugating with antibodies [27], small molecules [52], or other aptamers [53]. To provide proof-of-concept in vivo, LNCaP cell implanted mice (via intraperitoneal injection) were injected with SPIONs with or without PSMA ligand intravenously via tail vein (20 mg Fe/kg, Figure 5C). MR images were taken before injection and 1, 3, 6, 12 and 24-h post-injection. The injection dosage and monitoring time were set by referring to related research [42,54] and validating in our preliminary experiments. For reasons of safety, it is necessary to sterilize the nanoparticles before the injection, and filtration with Millipore filter (0.22 μm pore size) was performed for sterilization without altering the chemical or physical characteristics of the nanoparticles [55]. In addition, preventing contamination during the preparation process could be a practical way of producing endotoxin-free particles for in vivo assays. In Figure 5A, significant darkening was observed at the xenograft site (red circles) in tomographic images taken in 1 h, compared with that in mice injected with non-PSMA targeting SPIONs Fe_3_O_4_@DPA-PEG-NH_2_. When the noise-signal ratio at the xenograft site at different times were converted into a line chart (Figure 5B), negative contrast at 6 h was most significantly exhibited and faded along with time. At the time point of 6 h, no clearly appreciable contrast caused by non-specific binding SPIONs distributed in xenograft reign of LNCaP tumor-bearing mice could be noticed. In light of this, it was reasonable to assume that the SPIONs labeled with PSMA targeting ligand (Fe_3_O_4_@DPA-PEG-PSMA-1) could specifically accumulate in PSMA positive tissue and produce clear negative contrast. The darkening effect as a result of the SPIONs facilitated the visualization of the prostate.

#### 3.3.2. Histological Analysis

**Figure 6:**
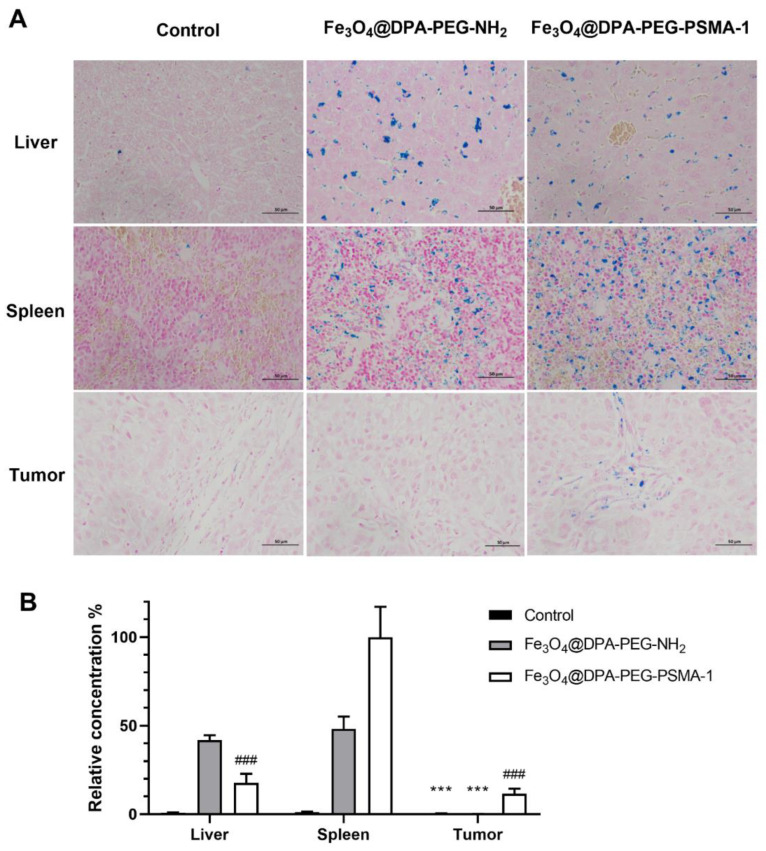
(**A**) Prussian blue and nuclear fast red double staining images of liver, spleen, and tumor tissues of Control group, Fe_3_O_4_@DPA-PEG-NH_2_ group and Fe_3_O_4_@DPA-PEG-PSMA-1 group 24 h after single-dose injection of PSMA-targeted and non-targeted SPIONs (Fe, 20 mg/kg) in PCa xenografted nude mice. Scale bars equal 50 μm. (**B**) Semi-quantification of relative concentration of SPIONs in liver, spleen, and tumor tissues of each group. Data are presented as means ± SD. Compared with Fe_3_O_4_@DPA-PEG-PSMA-1 in tumor tissue, *** *p* < 0.001. Compared with Fe_3_O_4_@DPA-PEG-PSMA-1 in spleen tissues, ^###^ *p* < 0.001. N = 6 per group.

In order to validate the presence of targeted SPIONs in mice, xenograft tumors were harvested and stained. Prussian blue staining of resected tumors from targeted SPIONs mice showed detectable residual SPIONs in the tumor tissues, even 24 h post-injection, compared with tissues from the control group. No appreciable accumulation of iron was noticed within the tumor of mice treated with non-PSMA-targeted Fe_3_O_4_@DPA-PEG-NH_2_. (Figure 6) We also assessed the iron accumulation in the livers and spleens. In contrast, iron deposition was found in the liver and spleen tissue of mice injected with targeting or non-targeting SPIONs (Figure 6). These results were consistent with SPIONs conjugated with PSMA-targeting polypeptides [20] or antibody [50] in previous research. The iron nanoparticles were supposed to be taken up and degraded intracellularly by macrophages in the liver and spleen [56,57]. These results suggested that Fe_3_O_4_@DPA-PEG-PSMA-1 might facilitate specific enhancement of prostate tumor tissue by targeting PSMA and could be metabolized and excreted, which may reduce accumulation in tissue and possible toxicity.

## 4. Conclusions

In summary, we developed SPIONs labelled with PSMA-targeting small molecules as a novel negative contrast agent that can effectively enhance PCa for MRI imaging in vivo. The Glu-Urea-Lys scaffold, the PSMA-targeting ligand adopted in our probe, mimics the natural ligand of PSMA. The probe demonstrated high binding affinity to PSMA and specific uptake by PSMA expressing prostate cancer cells. In a xenograft PCa mouse model, significant negative contrast could be specifically produced by the tracer, exhibiting the potential to exclusively enhance magnetic resonance detection of PCa. Nevertheless, the pharmacokinetic stability of the probe in vivo, and accumulation capability in target tumor tissue, which both contribute to imaging contrast of prostate tumor, will serve as prior concerns before the implementation of the SPIONs into clinical practice. Furthermore, work will continue with the probe to explore the potential capability to grade and stage the PCa tumor and predict its recurrence, serving as an early indicator of tumor progression.

## Figures and Tables

**Table 1:** Zeta potential of SPIONs.

Sample	Zeta Potential (mV)	
	pH7.4 PBS	FBS
Fe_3_O_4_ nanoparticles	−25.2 ± 0.3	−23.0 ± 0.9
Fe_3_O_4_@DPA-PEG-NH_2_	18.8 ± 0.7	19.2 ± 1.2
Fe_3_O_4_@DPA-PEG-PSMA-1	−22.4 ± 0.4	−17.4 ± 0.7

Note: Data are represented in mean and standard deviations (SD); PBS, phosphate buffered saline; FBS, fetal bovine serum.

**Table 2:** PSMA inhibitory activity.

Compound	*K*_i_
PSMA-1	2.4 ± 0.2 nM
Fe_3_O_4_@DPA-PEG-NH_2_	>100 μg(Fe)/mL
Fe_3_O_4_@DPA-PEG-PSMA-1	0.38 ± 0.08 μg(Fe)/mL

## Data Availability

The data are available on reasonable request from the corresponding authors.

## Supplementary Materials

The following are available online at www.mdpi.com/article/10.3390/pharmaceutics14102051/s1. Supplementary Figure S1: The T_2_ weighted imaging of Fe_3_O_4_@DPA-PEG-PSMA-1 of different concentrations (0.0375–0.6 μg(Fe)/mL) in PBS; Supplementary Scheme S1: The synthesis of PSMA-1. Supplementary Figure S2: ^1^H and ^13^C NMR spectra of PSMA-1 and intermediates; Supplementary Figure S3: HRMS spectra of PSMA-1 and intermediates.
