# Supplementary material for: Glu-Urea-Lys Scaffold Functionalized Superparamagnetic Iron Oxide Nanoparticles Targeting PSMA for In Vivo Molecular MRI of Prostate Cancer
Source: Pharmaceutics. 2022 Sep 26;14(10):2051. doi: 10.3390/pharmaceutics14102051 (PMC9608448; doi:10.3390/pharmaceutics14102051)

Supplementary Information

Glu-Urea-Lys Scaffold Functionalized Superparamagnetic Iron Oxide Nanoparticles targeting PSMA for In Vivo Molecular MRI of Prostate Cancer

Content

1. The T_2 weighted imaging of Fe_3O_4@DPA-PEG-PSMA-1 in PBS
2. ^{1}H and ^{13}C NMR spectra of PSMA-1 and intermediates
3. HRMS spectra of PSMA-1 and intermediates

1. The T2 weighted imaging of Fe3O4@DPA-PEG-PSMA-1 in PBS

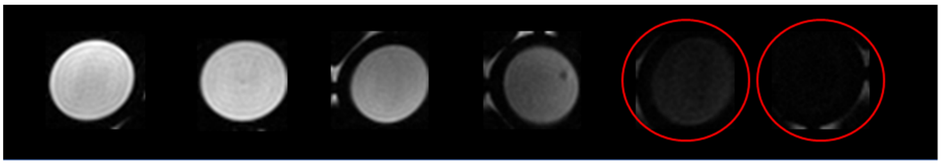

Supplementary Figure S1. The T_2 weighted imaging of Fe_3O_4@DPA-PEG-PSMA-1 of different concentrations (0.0375-0.6 μg(Fe)/ml) in PBS.

2. ^{1}H and ^{13}C NMR spectra of PSMA-1 and intermediates

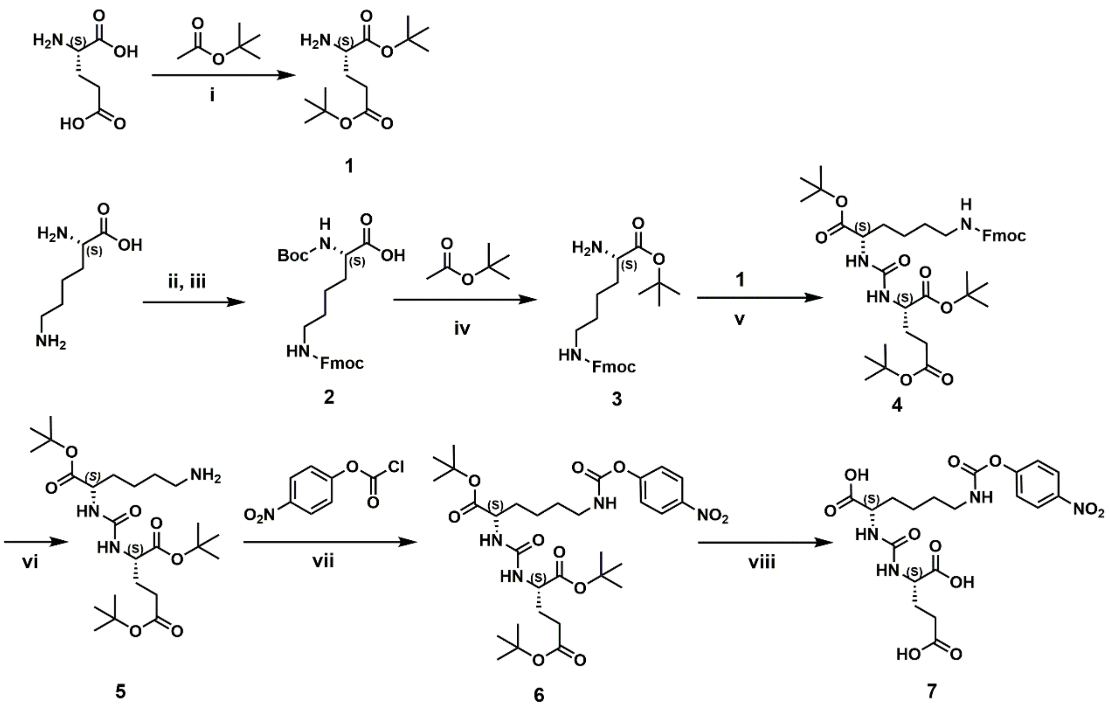

Supplementary Scheme S1. The synthesis of PSMA-1. Reagents and conditions: (i) 70% $HClO_4$, 47.6%; (ii) Fmoc-OSU, $CuSO_4$, $NaHCO_3$; (iii) Na_2CO_3, 8-hydroxyquinoline, Boc_2O, 64% in 2 steps; (iv) 70% $HClO_4$, 70%; (v) triphosgene, DIEA, 75.3%; (vi) DEA, 70%; (vii) DEA,CH_2Cl_2, 73%; (viii) 25%TFA/CH_2Cl_2, 85%.

*di-tert-butyl L-glutamate (**1**)*

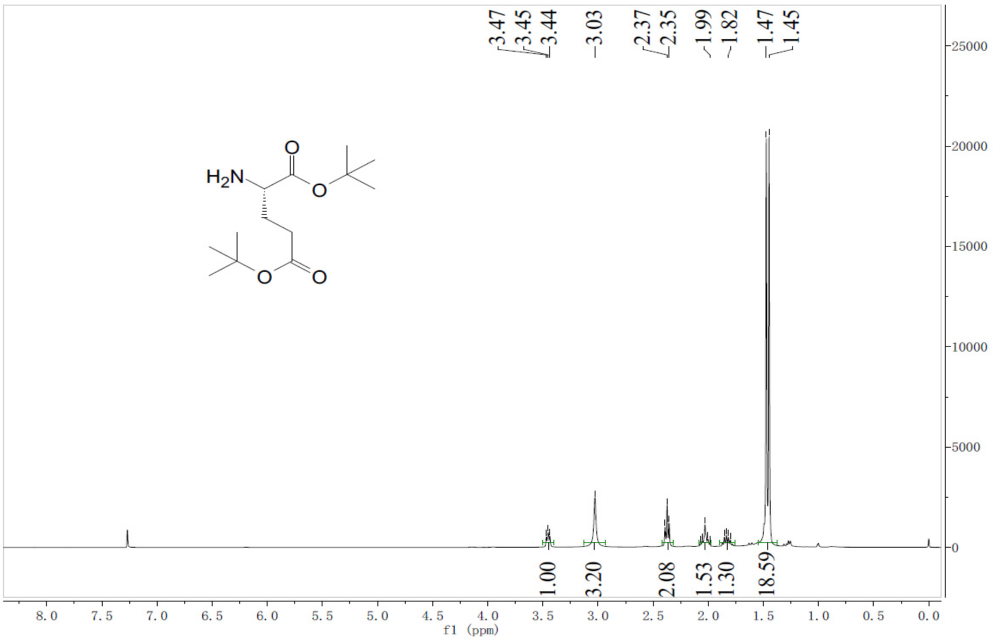

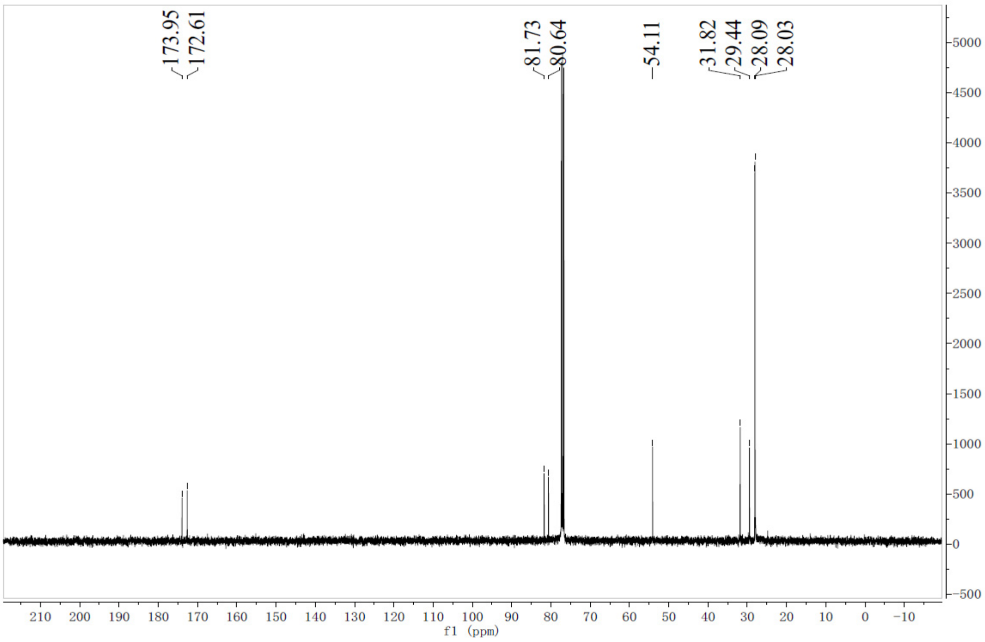

N[6]*-Fmoc-N*[2]*-Boc-L-lysine* (***2***)

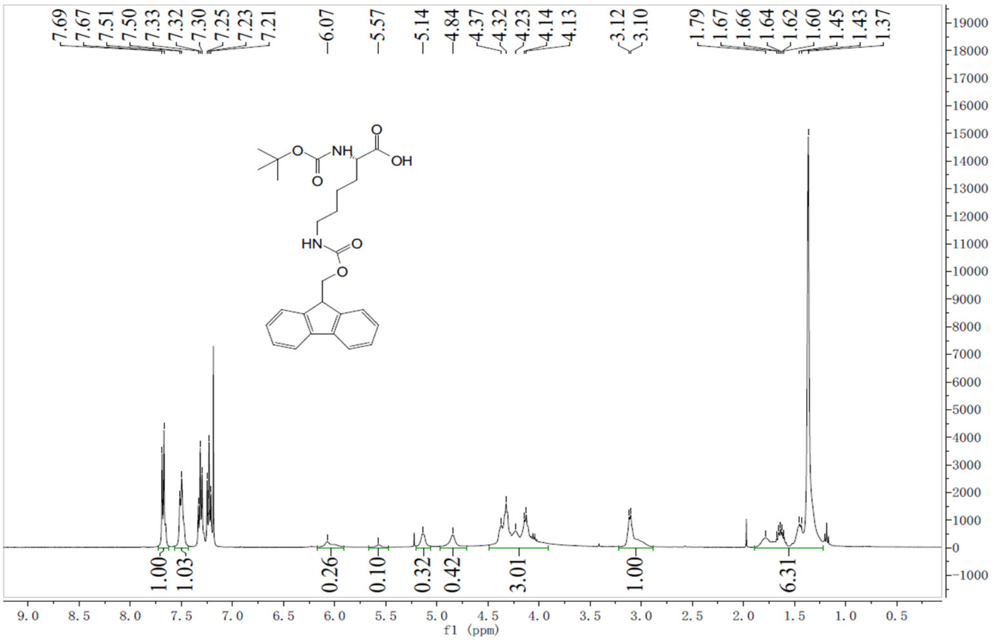

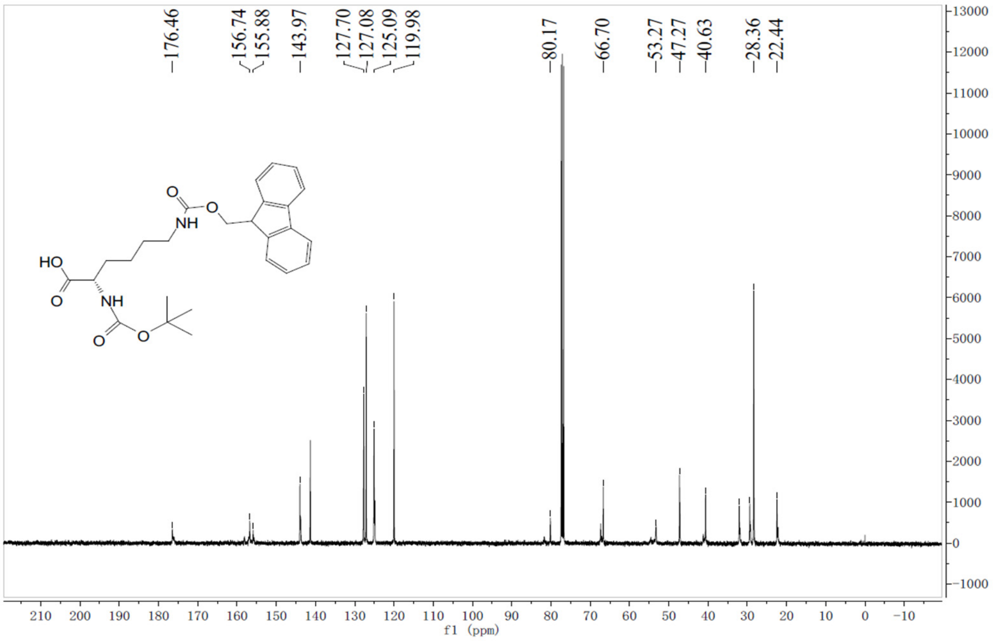

*tert-butyl N[6]-Fmoc-L-lysinate (**3**)*

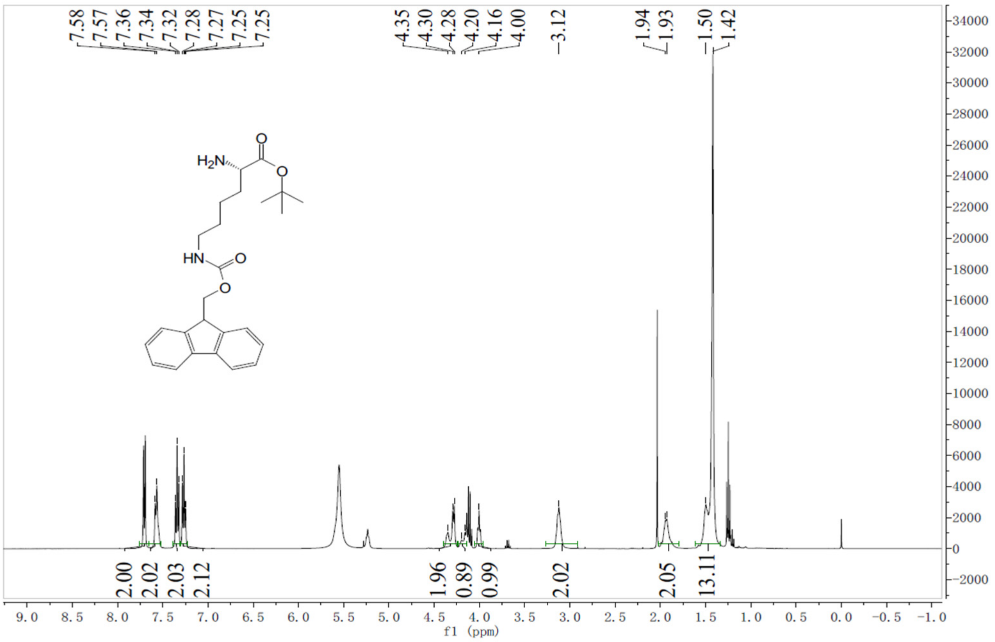

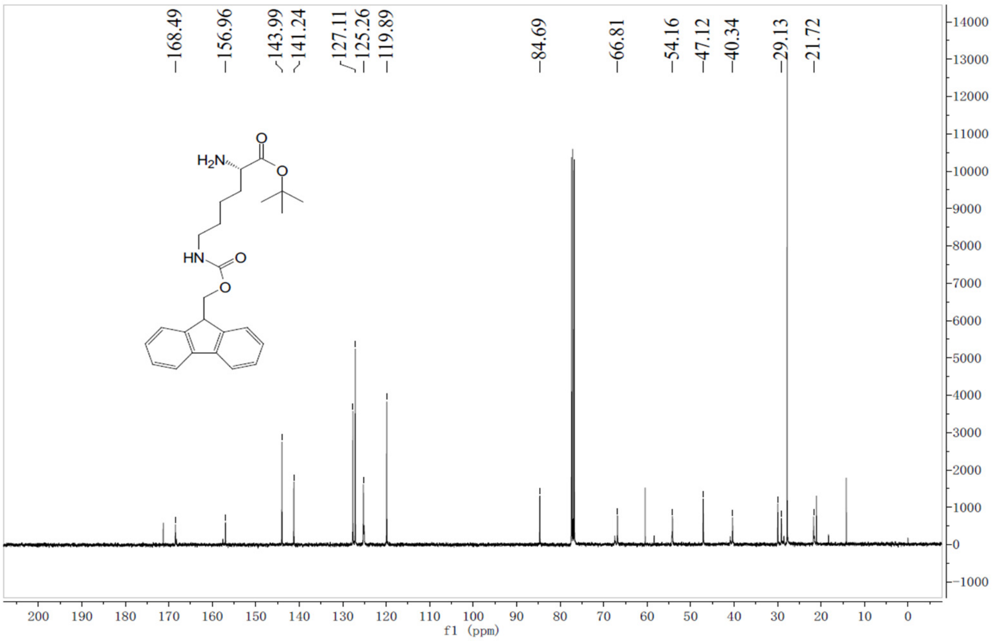

*(Tert-butyl-N^6-Fmoc)-L-Lys-Urea-(di-tert-butyl)-L-Glu (**4**)*

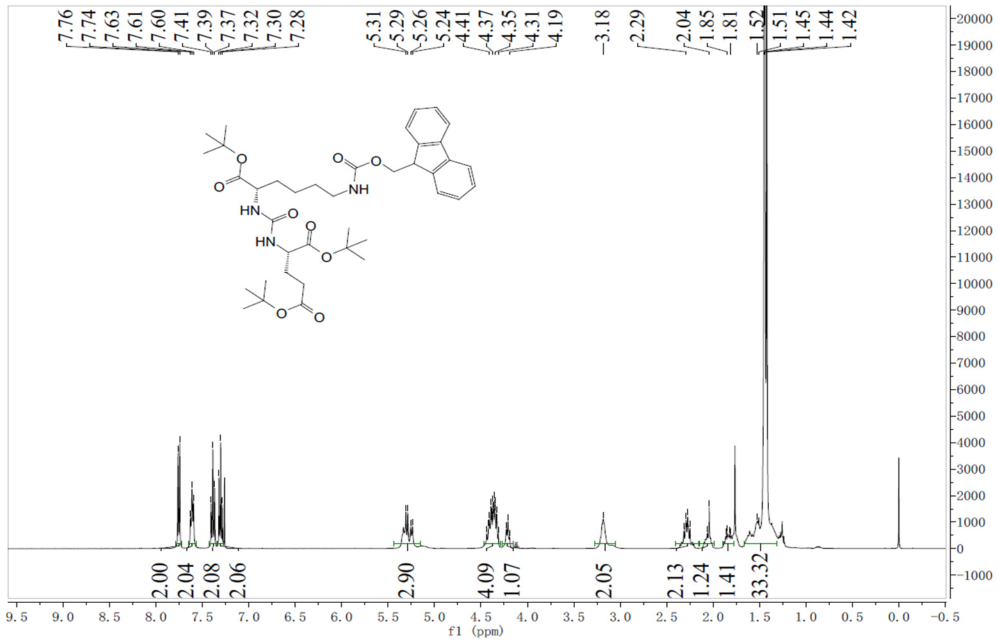

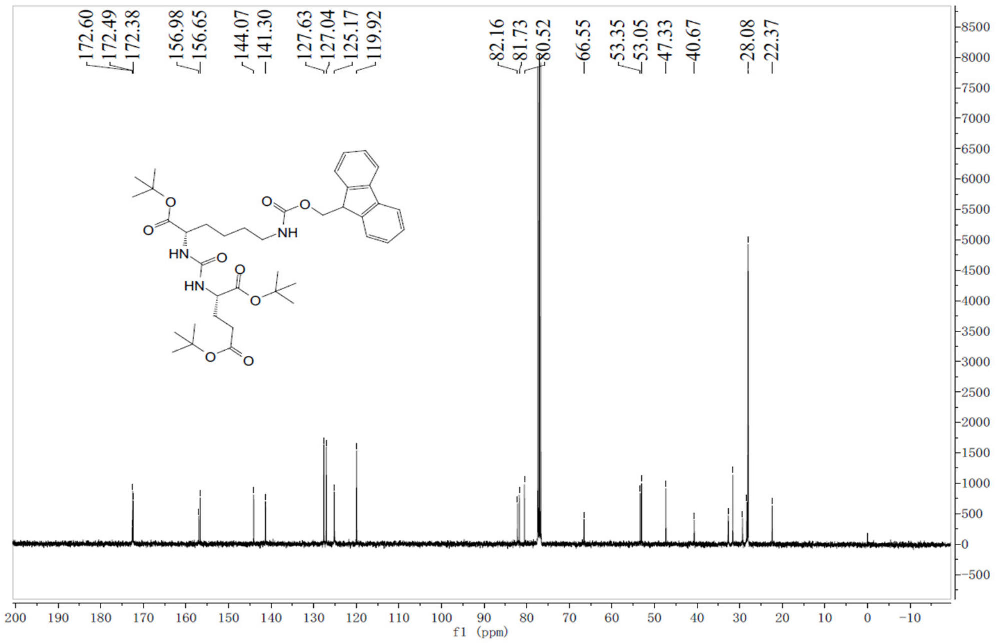

*4.2.5. (Tert-butyl)-L-Lys-Urea-(di-tert-butyl)-L-Glu (**5**)*

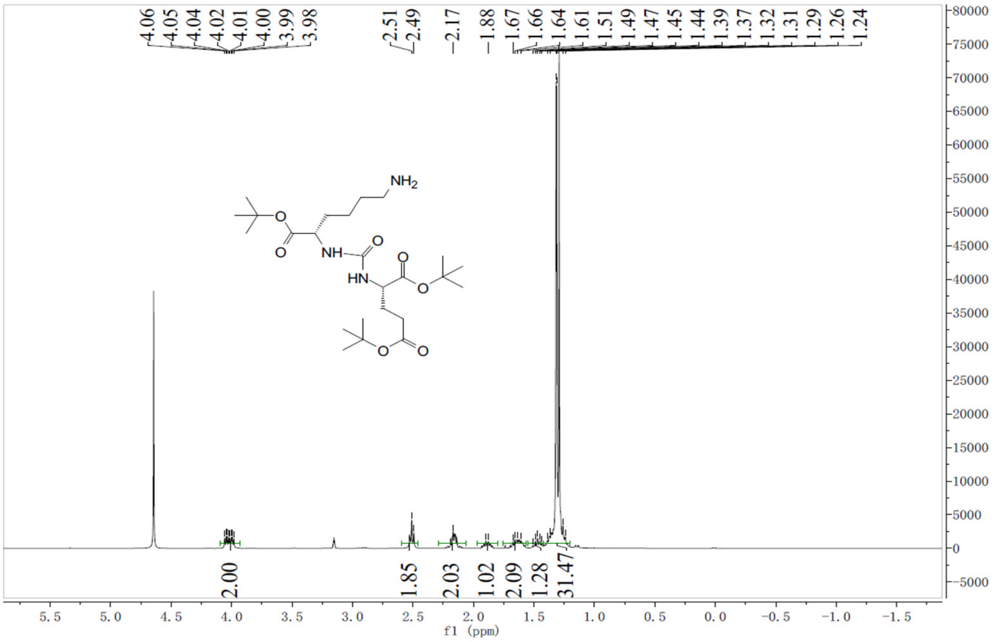

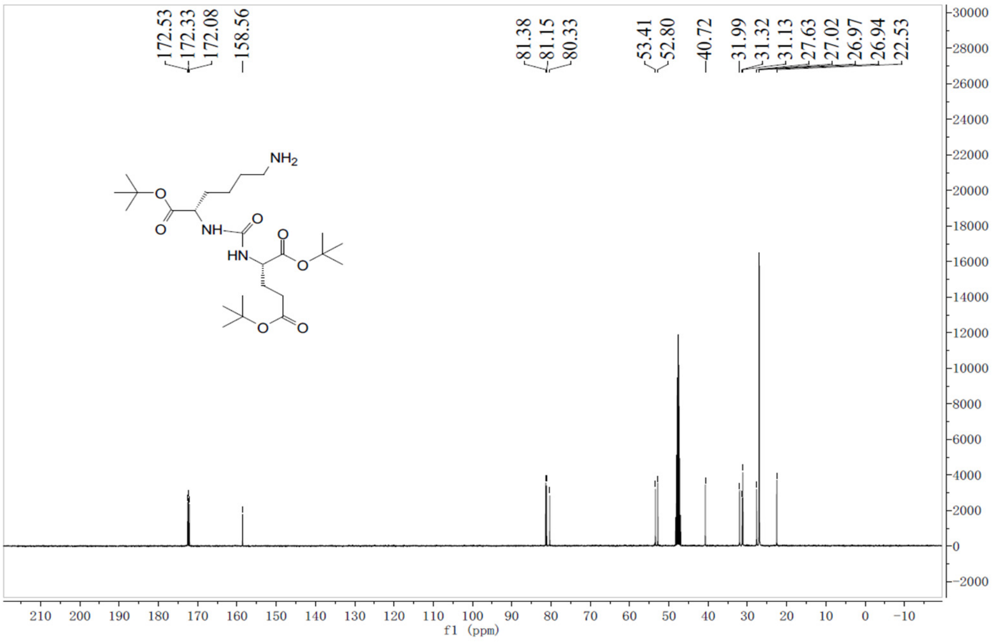

*di-tert-butyl (((S)-1-(tert-butoxy)-6-(((4-nitrophenoxy)carbonyl)amino)-1-oxohexan-2-yl) carbamoyl)-L-glutamate (**6**)*

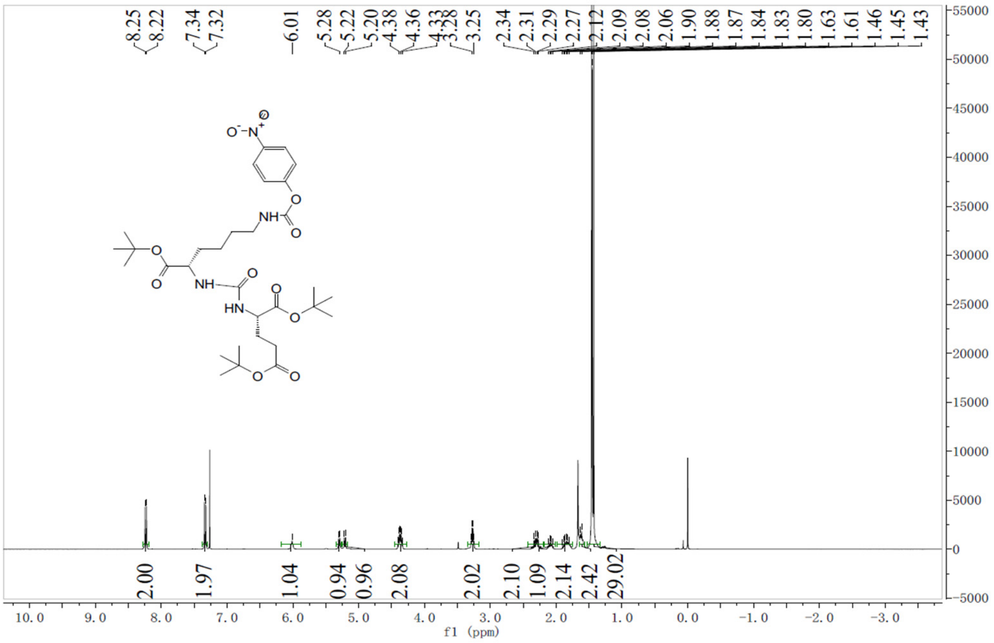

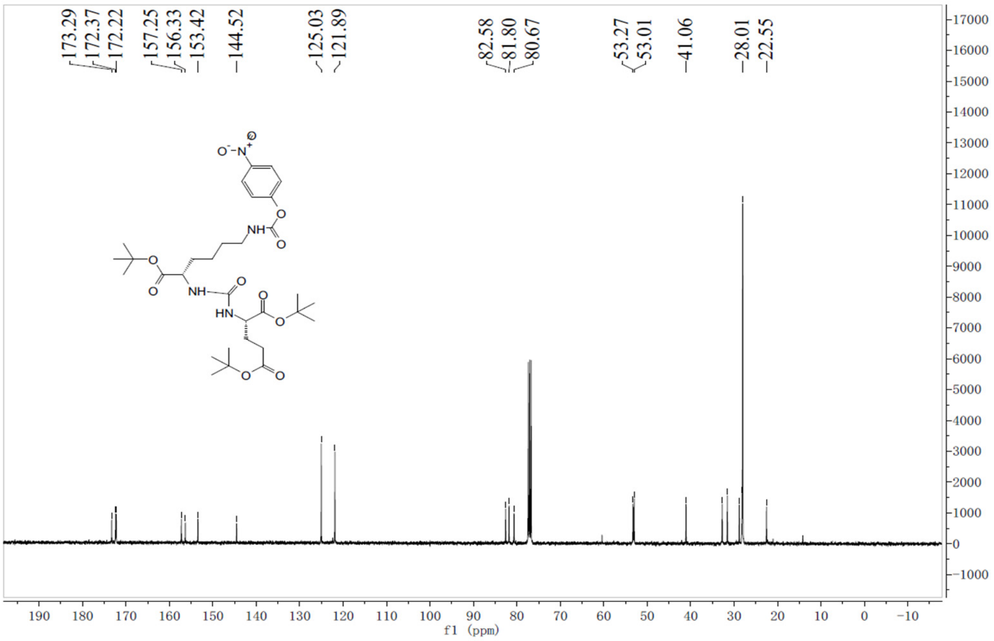

*(((S)-1-carboxy-5-(((4-nitrophenoxy)carbonyl)amino)pentyl)carbamoyl)-L-glutamic acid (**PSMA-1**)*

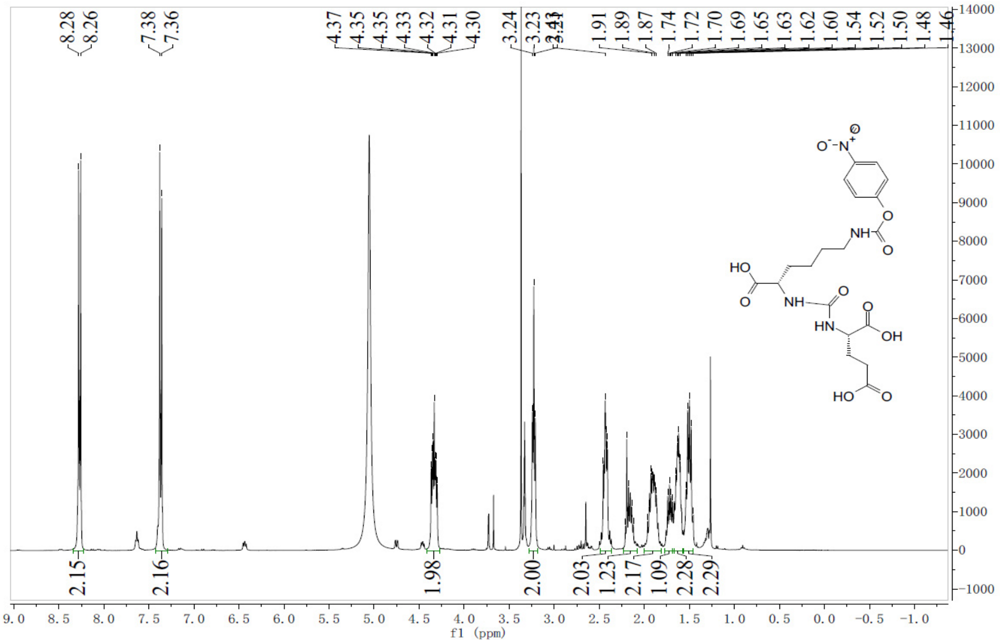

^{1}H NMR of Compound 3-007

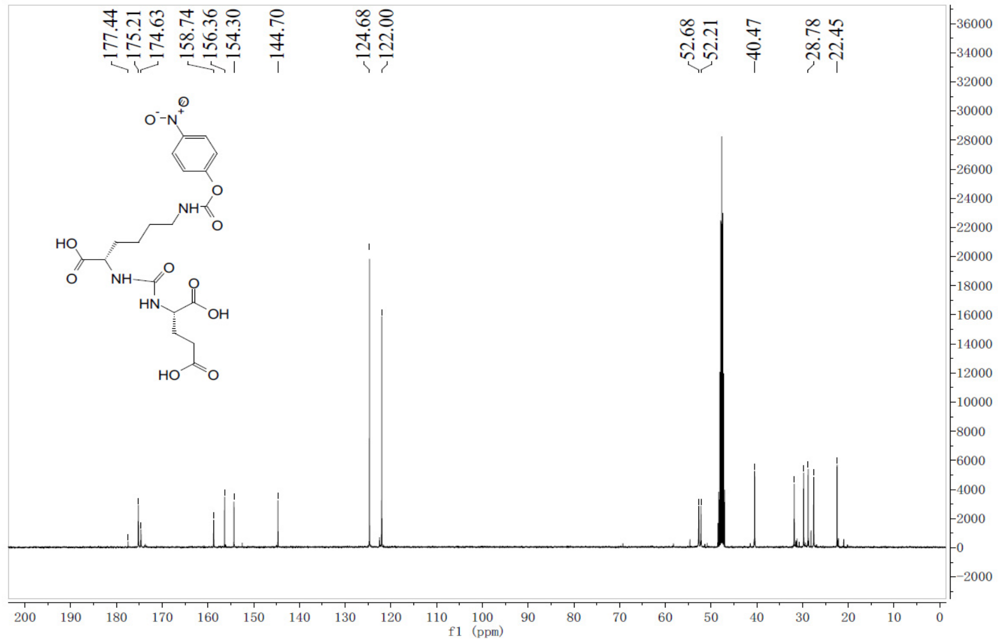

3. HRMS spectra of PSMA-1 and intermediates

di-tert-butyl L-glutamate (**1**)

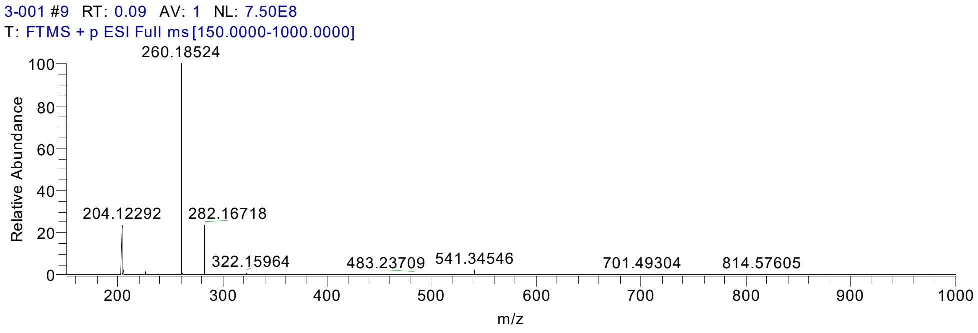

N^6-Fmoc-N^2-Boc-L-lysine (**2**)

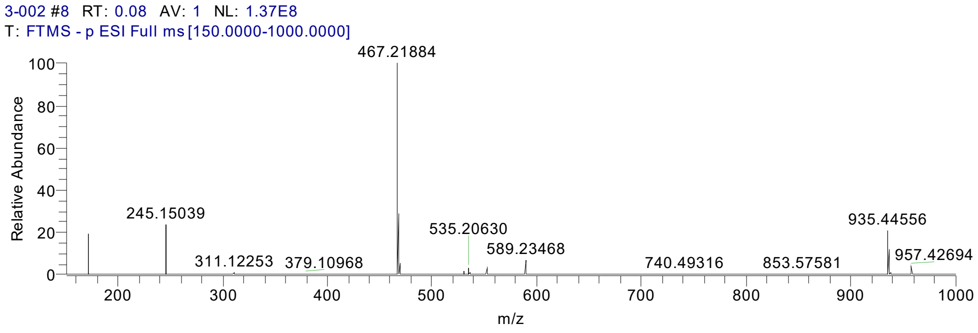

tert-butyl N^6-Fmoc-L-lysinate (**3**)

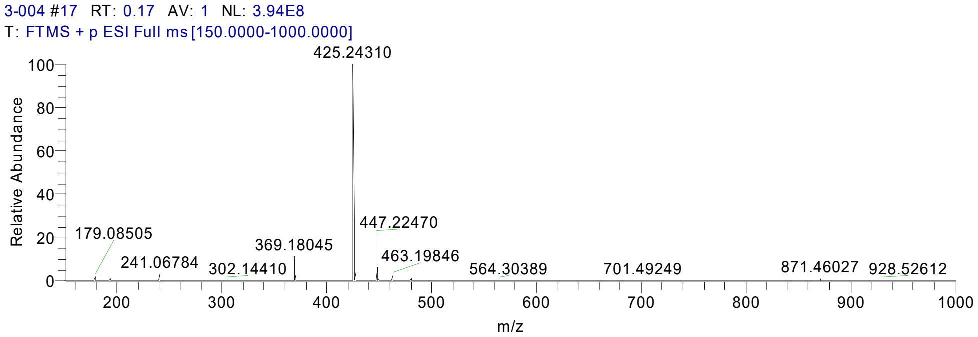

*(Tert-butyl-N^6-Fmoc)-L-Lys-Urea-(di-tert-butyl)-L-Glu (**4**)*

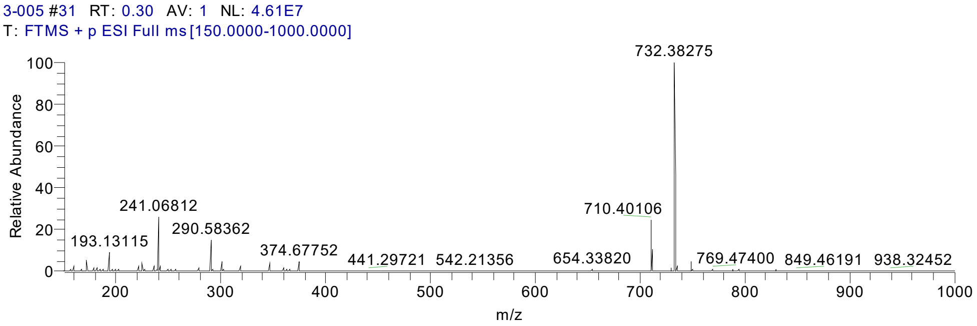

*(Tert-butyl)-L-Lys-Urea-(di-tert-butyl)-L-Glu (**5**)*

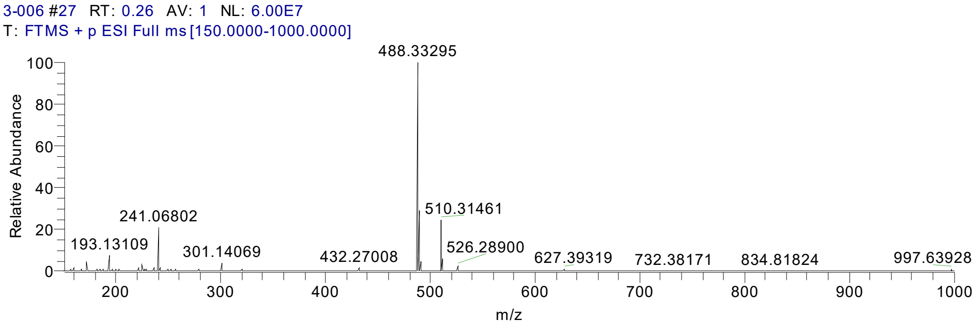

*di-tert-butyl (((S)-1-(tert-butoxy)-6-(((4-nitrophenoxy)carbonyl)amino)-1-oxohexan-2-yl) carbamoyl)-L-glutamate (**6**)*

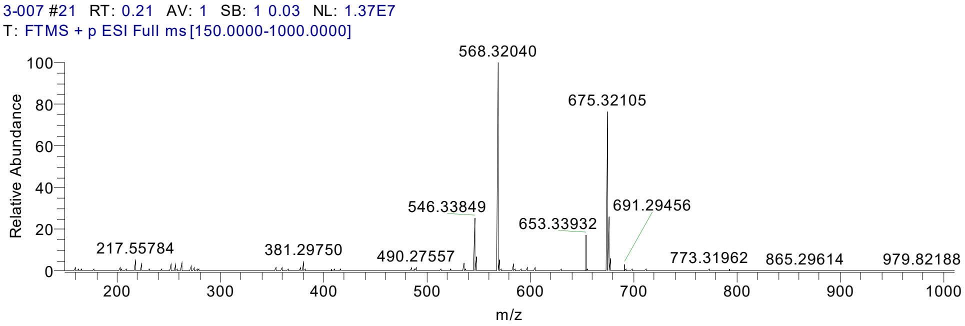

*(((S)-1-carboxy-5-(((4-nitrophenoxy)carbonyl)amino)pentyl)carbamoyl)-L-glutamic acid (**PSMA-1**)*

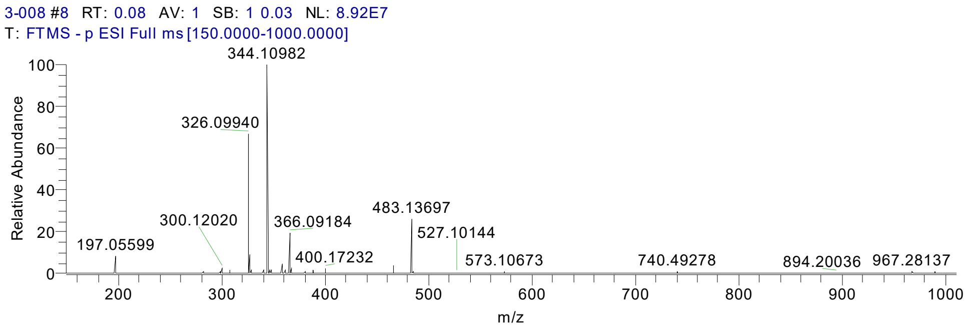

Supplement: Supplementary file 1 [file pharmaceutics-14-02051-s001.zip › pharmaceutics-1849882-supplementary.pdf]
